# Axonal Endoplasmic Reticulum Dynamics and Its Roles in Neurodegeneration

**DOI:** 10.3389/fnins.2020.00048

**Published:** 2020-01-29

**Authors:** Zeynep Öztürk, Cahir J. O’Kane, Juan José Pérez-Moreno

**Affiliations:** Department of Genetics, University of Cambridge, Cambridge, United Kingdom

**Keywords:** endoplasmic reticulum, hereditary spastic paraplegia, axonal transport, neurodegeneration, smooth ER, organelle contact sites, calcium stores

## Abstract

The physical continuity of axons over long cellular distances poses challenges for their maintenance. One organelle that faces this challenge is endoplasmic reticulum (ER); unlike other intracellular organelles, this forms a physically continuous network throughout the cell, with a single membrane and a single lumen. In axons, ER is mainly smooth, forming a tubular network with occasional sheets or cisternae and low amounts of rough ER. It has many potential roles: lipid biosynthesis, glucose homeostasis, a Ca^2+^ store, protein export, and contacting and regulating other organelles. This tubular network structure is determined by ER-shaping proteins, mutations in some of which are causative for neurodegenerative disorders such as hereditary spastic paraplegia (HSP). While axonal ER shares many features with the tubular ER network in other contexts, these features must be adapted to the long and narrow dimensions of axons. ER appears to be physically continuous throughout axons, over distances that are enormous on a subcellular scale. It is therefore a potential channel for long-distance or regional communication within neurons, independent of action potentials or physical transport of cargos, but involving its physiological roles such as Ca^2+^ or organelle homeostasis. Despite its apparent stability, axonal ER is highly dynamic, showing features like anterograde and retrograde transport, potentially reflecting continuous fusion and breakage of the network. Here we discuss the transport processes that must contribute to this dynamic behavior of ER. We also discuss the model that these processes underpin a homeostatic process that ensures both enough ER to maintain continuity of the network and repair breaks in it, but not too much ER that might disrupt local cellular physiology. Finally, we discuss how failure of ER organization in axons could lead to axon degenerative diseases, and how a requirement for ER continuity could make distal axons most susceptible to degeneration in conditions that disrupt ER continuity.

## Introduction

**FIGURE 1:**
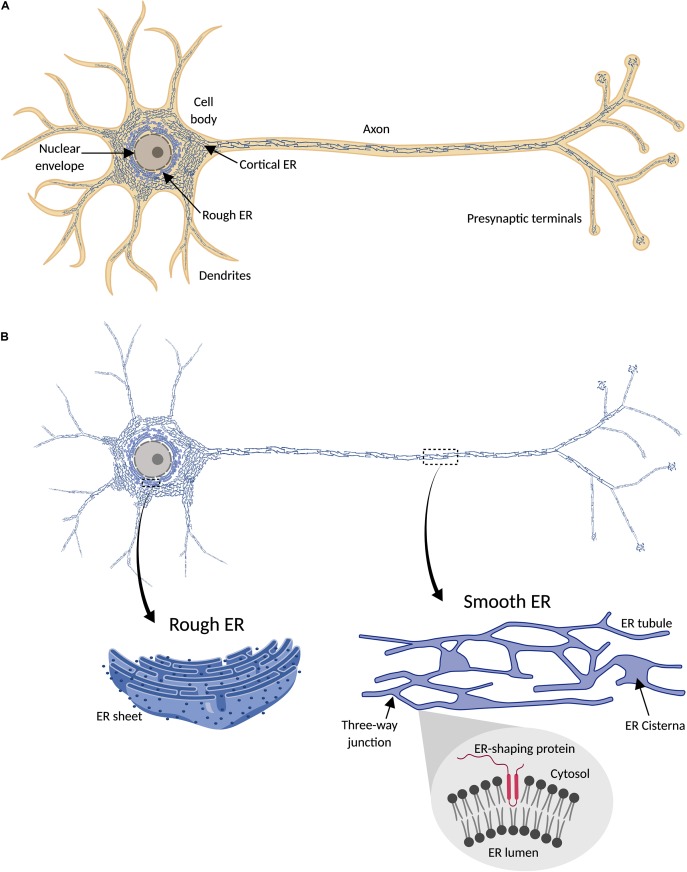
Endoplasmic reticulum (ER) distribution in neuronal cells. **(A)** ER network (blue) is continuously distributed throughout the cytosol (orange) in neurons, including cell body, axon, presynaptic terminals, and most dendrites. **(B)** Since ER forms a continuous structure that follows the shape of the cell, it has been termed “a neuron within a neuron” (Berridge, 1998, 2002). In the cell body, continuous with the nuclear envelope, is found rough ER, characterized by its sheet-like morphology and the presence of ribosomes (dark blue points) attached to the ER membrane (magnified detail on the left). Peripheral ER (smooth) includes cortical ER in the cell body and dendritic and axonal ER, and is mainly formed by a network of interconnected tubules, with occasional sheets or cisternae (magnified detail on the right). Tubulation of ER membrane is physically promoted by ER-shaping proteins, which share a characteristic intramembrane domain inserted in the cytosolic face of the ER membrane (magnified detail on the bottom right).

Endoplasmic reticulum (ER) is a membrane-bound organelle found throughout the cytoplasm of all eukaryotic cells. It typically comprises more than half of the total animal cell membranes (Alberts et al., 2002), or 35% of cytoplasmic volume at any one time (Valm et al., 2017). Light and electron microscopy (EM), and membrane dye labeling, suggest that uniquely among organelles, the ER appears pervasive and continuous throughout cells, including neurons (Tsukita and Ishikawa, 1976; Terasaki et al., 1994; Wu et al., 2017; Yalçın et al., 2017) (Figure 1). Physically connected to the nuclear envelope, and sharing with it a common lumen, ER includes rough and smooth ER domains. The first is called “rough” due to the density of ribosomes on its surface, which are involved in synthesis of proteins for export from the cytosol. Rough ER is found mainly around the nucleus, organized in a network of membrane sheets that envelop a lumen with a constant distance between sheets, and interconnected by spiral ramps (Terasaki et al., 2013). Smooth ER, with few ribosomes, is found in peripheral parts of the cell, as a network of interconnected cylindrical tubules, with occasional and irregularly spaced sheets (with a constant lumen size like rough ER) or cisternae with a larger and less regularly shaped lumen. This variability in ER morphology influences ER function. Tubules are regions for lipid synthesis and contacts with other organelles; cisternae, with a larger lumen, in addition have more capacity for calcium storage (Schwarz and Blower, 2016). Therefore, the extent and spatial distribution of different ER morphologies depends on cell type and cell demands.

Rough ER is specialized for protein export from the cytosol. Most exported proteins have an N-terminal signal sequence that directs them to rough ER, where they are co-translationally transported across the ER membrane, folded in the ER lumen, and sorted for transport to another intracellular membrane compartment or the extracellular secretory pathway. Type I transmembrane proteins, with an extracellular or luminal N-terminus, follow similar pathways, but remain embedded in the ER membrane, due to an internal transmembrane domain that prevents the rest of the protein from being exported (Schwarz and Blower, 2016). Smooth ER, on the other hand, plays important roles in both lipid (Jacquemyn et al., 2017) and glucose (Marini et al., 2016; Müller et al., 2018) metabolism, as well as in Ca^2+^ dynamics (Schwarz and Blower, 2016).

Until recently, axonal ER received scant attention since its characterization by electron microscopy some four decades ago e.g., Tsukita and Ishikawa (1976), partly due to the lack of molecular markers and the difficulty of resolving it using conventional osmium staining in thin EM sections. However, both these hurdles have recently been overcome e.g., Villegas et al. (2014) and Yalçın et al. (2017). Axonal ER has the characteristics of a largely smooth tubular ER network, which must share many of its functions with the better characterized equivalent network in non-neuronal cells, but with specialized roles and constraints that reflect the long and narrow dimensions of axons. In this review, we wish to focus on the behavior and function of axonal ER, drawing both on work in axons, but also examining how our broader knowledge of the role of smooth tubular ER might apply to neurons, particularly axons. As background to our review, we have summarized proteins with roles related to tubular ER function, and which of them have been observed in axons (Table 1).

**TABLE 1:** Functional classification of tubular ER-related proteins and localization in axons.

***Protein***	***Function***	***Axonal localization***
**Tubular ER Morphogenesis**		
ARL6IP1	ER tubulation (Yamamoto et al., 2014; **Dong et al., 2018**)	Yes (Dong et al., 2018)
Ataxin-2	Tubular ER network organization (**del Castillo et al., 2019**)	Yes (Ralser et al., 2005)
ATLs	Homotypic membrane fusion between ER tubules (Hu et al., 2009; **Orso et al., 2009**)	Yes (Zhu et al., 2003; Lee et al., 2009; Park et al., 2010; Behrendt et al., 2019; Krols et al., 2019)
INPP5K	ER tubulation (**Dong et al., 2018**)	Yes (Dong et al., 2018)
Lunapark	Stabilization of three-way junctions between ER tubules (Chen et al., 2012, 2015)	Yes (Breuss et al., 2018)
MCTP2/Pex30	ER tubulation (Joshi et al., 2016, 2018)	Yes (Genç et al., 2017)
MFN2	Control of ER network continuity (de Brito and Scorrano, 2008)	Yes (Cagalinec et al., 2013)
Protrudin	ER tubulation (Chang et al., 2013)	Yes (Pantakani et al., 2011)
Rab10	Tubular ER network organization (English and Voeltz, 2013)	Yes (Deng et al., 2014; Wang et al., 2011; Sasidharan et al., 2012; Xu et al., 2014)
Rab18	Tubular ER network organization (Gerondopoulos et al., 2014)	Unknown
Rab3GAPs	Tubular ER network organization by targeting Rab18 to the ER (Gerondopoulos et al., 2014)	Yes (Müller et al., 2011)
REEPs	ER tubulation (Voeltz et al., 2006; **Beetz et al., 2013**; **Yalçın et al., 2017**)	Yes (Park et al., 2010; Yalçın et al., 2017)
RTNs	ER tubulation (Voeltz et al., 2006; **O’Sullivan et al., 2012**; Rämö et al., 2016; Wang S. et al., 2016; **Yalçın et al., 2017**)	Yes (O’Neill et al., 2004; O’Sullivan et al., 2012; Deng et al., 2013; Levitan et al., 2016; Rao et al., 2016; Yalçın et al., 2017)
VAPA/B	Control of ER network continuity (**Lindhout et al., 2019**)	Yes (Pennetta et al., 2002; Kirmiz et al., 2018; Gómez-Suaga et al., 2019; Lindhout et al., 2019)

**Tubular ER Motility and Association With MTs**		

P180	MT stabilization from ER tubules (**Farías et al., 2019**)	Yes (Farías et al., 2019)
p600	MT interaction and stabilization, and interaction with ER tubules (**Shim et al., 2008**)	Yes (Shim et al., 2008)
Rab10	Regulation of ER tubules extension along MTs (English and Voeltz, 2013)	Yes (Wang et al., 2011; Sasidharan et al., 2012; Deng et al., 2014; Xu et al., 2014)
REEP1	Interaction between tubular ER and MTs (Park et al., 2010)	Yes (Park et al., 2010)
RTNs	Constriction of ER tubules (Espadas et al., 2019)	Yes (O’Neill et al., 2004; O’Sullivan et al., 2012; Deng et al., 2013; Yalçın et al., 2017)
Sec61β	Subunit of the Sec61 translocon complex that interacts with MTs (Zhu et al., 2018)	Yes (Summerville et al., 2016; Farías et al., 2019; Lindhout et al., 2019)
Spastin	ATP-dependent severing of MTs (**Sherwood et al., 2004; Trotta et al., 2004;** Roll-Mecak and Vale, 2005; Park et al., 2010)	Yes (Trotta et al., 2004; Park et al., 2010)
STIM1	MT-mediated transport of ER tubules by interacting with EB1 (Grigoriev et al., 2008; Pavez et al., 2019)	Yes (Mitchell et al., 2012; Shim et al., 2013; de Juan-Sanz et al., 2017; Pavez et al., 2019)
VAPA/B	Regulation of ER tubules dynamics (**Lindhout et al., 2019**)	Yes (Pennetta et al., 2002; Kirmiz et al., 2018; Gómez-Suaga et al., 2019; Lindhout et al., 2019)

**Tubular ER Turnover**		

ATL3	Turnover of ER tubules via autophagy by interacting with the ATG8 family protein GABARAP (Chen et al., 2019)	Yes (Behrendt et al., 2019; Krols et al., 2019)
RTN3	Turnover of ER tubules via autophagy by interacting with ATG8 family proteins LC3s/GABARAPs (Grumati et al., 2017)	Yes (Kumamaru et al., 2004; Deng et al., 2013)
TEX264	Turnover of tubular ER three-way junctions via autophagy by interacting with ATG8 family proteins LC3s/GABARAPs (An et al., 2019; Chino et al., 2019)	Unknown

**Tubular ER-Endosome MCSs**		

Spastin	Endosomal tubule fission at ER-endosomes MCSs (**Allison et al., 2013, 2017**)	Yes (Trotta et al., 2004; Park et al., 2010)
Protrudin	ER-late endosomes tethering with VAPA (Raiborg et al., 2015)	Yes (Pantakani et al., 2011)
PTP1B	Phosphorylation of EGFR at ER-endosomes MCSs (Eden et al., 2010)	Unknown
Vps13C	Lipid transfer protein that mediates ER-endosome/lysosomes contacts (Kumar N. et al., 2018)	Unknown
Osbp-related proteins	Lipid transfer protein ORP1L mediates ER-late endosomes tethering by interacting with Rab7 and VAP (Rocha et al., 2009) Lipid transfer protein Osbp associates with ER-endosomes MCSs (Dong et al., 2016) Lipid transfer protein ORP5 mediates ER-endosome tethering by interacting with NPC1 (Du et al., 2011)	Yes (Ma et al., 2012)
PDZD8	Rab7-dependent interaction between ER and late endosomes/lysosomes (Guillén-Samander et al., 2019)	Unknown
VAPA/B	ER-endosomes tethering by interacting with SNX2 and lipid transfer protein Osbp (Dong et al., 2016) ER-late endosomes tethering via Rab7 and lipid transfer protein ORP1L (Rocha et al., 2009), Rab7 and protrudin (Raiborg et al., 2015), or lipid transfer protein STARD3 (Alpy et al., 2013; Wilhelm et al., 2017)	Yes (Pennetta et al., 2002; Kirmiz et al., 2018; Gómez-Suaga et al., 2019; Lindhout et al., 2019)

**Tubular ER-Mitochondria MCSs**		

grp75	Chaperone that mediates the interaction between IP3R and VDAC at ER-mitochondria MCSs (Szabadkai et al., 2006; **Honrath et al., 2017; Lee S. et al., 2019**)	Yes (Willis et al., 2005; Lee S. et al., 2019)
IP3R	Ca^2+^-release channel receptor that promotes ER-mitochondria tethering by interacting with VDAC (Cárdenas et al., 2010; **Bernard-Marissal et al., 2015**; Bartok et al., 2019; **Lee S. et al., 2019**)	Yes (Takel et al., 1992; Shim et al., 2008; Rao et al., 2016; Lee S. et al., 2019)
MFN2	ER-mitochondria tethering (de Brito and Scorrano, 2008)	Yes (Cagalinec et al., 2013)
Osbp-related proteins	Lipid transfer proteins ORP5 and ORP8 associates with ER-mitochondria MCSs (Rochin et al., 2019)	Yes (Ma et al., 2012)
REEP1	Formation of ER-mitochondria MCSs (Lim et al., 2015)	Yes (Park et al., 2010)
PDZ8	ER-mitochondria tethering (**Hirabayashi et al., 2017**)	Unknown
SigR1	Chaperone that promotes IP3R-VDAC interaction at ER-mitochondria MCSs by stabilizing IP3R (Hayashi and Su, 2007)	Yes (Prause et al., 2013)
VAPA	Interaction with the lipid transfer protein Vps13A at ER-mitochondria MCSs (Yeshaw et al., 2019)	Yes (Kirmiz et al., 2018; Lindhout et al., 2019)
VAPB	ER-mitochondria tethering by interacting with PTPIP51 (De Vos et al., 2012; Stoica et al., 2014; **Gómez-Suaga et al., 2019**)	Yes (Gómez-Suaga et al., 2019; Lindhout et al., 2019)
Vps13A	Lipid transport protein that interact with VAPA at ER-mitochondria MCSs (Kumar N. et al., 2018; Yeshaw et al., 2019)	Unknown

**Tubular ER-PM MCSs**		

E-Syts	Ca^2+^-dependent lipid transfer protein at ER-PM MCSs (Schauder et al., 2014; Yu et al., 2016)	Yes (Kikuma et al., 2017; Kim et al., 2018)
JPHs	ER-PM tethering (reviewed in **Landstrom et al., 2014**)	Unknown
Osbp-related proteins	Lipid transfer protein Osh3 interact with VAP (Stefan et al., 2011) and Sec22b (Petkovic et al., 2014) at ER-PM MCSs Lipid transfer proteins ORP5 and ORP8 associates with ER-PM MCSs (Chung et al., 2015) Lipid transfer protein ORP3 interact with VAPA at ER-PM MCSs (Weber-Boyvat et al., 2015)	Yes (Ma et al., 2012)
Sec22b	ER-PM tethering by interacting with Syntaxin1 **(Petkovic et al., 2014**) Interaction with lipid transfer proteins Osh2 and Osh3 (Petkovic et al., 2014)	Yes (Petkovic et al., 2014)
SigR1	Chaperone that attenuates of STIM1-Orai1 interaction at ER-PM MCSs by associating with STIM1 (Srivats et al., 2016)	Yes (Prause et al., 2013)
STIM1	Ca^2+^ sensor that controls of the store operated Ca^2+^ entry (SOCE) from the extracellular medium into the ER by interacting with the PM Ca^2+^ channel Orai1 (reviewed in Carrasco and Meyer, 2011) Regulation of PM Ca^2+^ uptake (**de Juan-Sanz et al., 2017**)	Yes (Mitchell et al., 2012; Shim et al., 2013; **de Juan-Sanz et al., 2017; Pavez et al., 2019**)
STIM2	Control of SOCE in neurons (**Berna-Erro et al., 2009**)	Unknown
TMEM24	Lipid transfer protein with Ca^2+^-dependent localization at ER-PM MCSs (**Sun et al., 2019**)	Yes (Sun et al., 2019)
VAPA/B	ER-PM tethering by interacting with Kv2 potassium channels (Johnson et al., 2018) or Osbp-related lipid transfer proteins (Stefan et al., 2011; Petkovic et al., 2014; Chung et al., 2015)	Yes (Pennetta et al., 2002; Kirmiz et al., 2018; Gómez-Suaga et al., 2019; Lindhout et al., 2019)

**Tubular ER Association with LDs/Peroxisomes**		

ATL1	Regulation of LDs size (Klemm et al., 2013)	Yes (Zhu et al., 2003; Park et al., 2010)
FATP1	Regulation of LDs growth by interacting with DGAT2 at ER-LDSs interface (Xu et al., 2012)	Unknown
LDAH	Regulation of LDs growth (Goo et al., 2017)	Yes (Yalçın et al., 2017)
MCTP2/Pex30	LD and peroxisome biogenesis (Joshi et al., 2018)	Yes (Genç et al., 2017)
NRZ complex	Part of the Rab18-NRZ/SNARE complex, which mediates ER-LD tethering and LD growth (Xu et al., 2018)	Unknown
Rab18	LDs growth by ER-LDs tethering (Martin et al., 2005; Ozeki et al., 2005; Xu et al., 2018)	Unknown
Rab3GAPs	ER-LDs tethering and LDs growth by activating and targeting Rab18 (Xu et al., 2018)	Yes (Müller et al., 2011)
REEP1	Regulation of LDs size (Falk et al., 2014)	Yes (Park et al., 2010)
Seipin	Determination of the site of LD formation and facilitation of lipid transfer from ER to LDs (Salo et al., 2019)	Unknown
Spastin	Regulation of LDs size (**Papadopoulos et al., 2015**)	Yes (Trotta et al., 2004; Park et al., 2010)
Syntaxin14	Regulation of LDs growth at ER-LDs contacts (Datta et al., 2019)	Unknown
SNAREs	Part of the Rab18-NRZ/SNARE complex, which mediates ER-LD tethering and LD growth (Xu et al., 2018)	Unknown
TBC1D20	LDs growth (Nevo-Yassaf et al., 2012; Liegel et al., 2013)	Unknown
VAPB	ER-peroxisomes tethering by interacting with ACBD5 (Costello et al., 2017)	Yes (Gómez-Suaga et al., 2019; Lindhout et al., 2019)
Vps13A	Lipid transfer protein that interact with VAPA at ER-LDs contacts (Kumar N. et al., 2018; Yeshaw et al., 2019)	Unknown
Vps13C	Lipid transfer protein that mediates ER-LDs contacts (Kumar N. et al., 2018)	Unknown

**Tubular ER Ca^2+^ Uptake/Release**		

IP3R	Ca^2+^-induced Ca^2+^ release to the cytosol (Straub et al., 2000; **Ross, 2012**)	Yes (Takel et al., 1992; Shim et al., 2008; Rao et al., 2016; Lee S. et al., 2019)
RyR	Ca^2+^-induced Ca^2+^ release to the cytosol (Mandikian et al., 2014; Villegas et al., 2014)	Yes (Kakizawa et al., 2007; Shimizu et al., 2008)
SERCA	ATPase pump that mediates ER Ca^2+^ uptake from the cytoplasm (reviewed in: Britzolaki et al., 2018; Carreras-Sureda et al., 2018)	Yes (Merianda et al., 2009)
SPoCK/ATP2C	ATPase pump that may mediate ER Ca^2+^ uptake from the cytoplasm (Southall et al., 2006)	Unknown

*Members of the same family protein are shown in the same row when they are generally related with the same function (for example RTNs in tubular ER morphogenesis); in the “*Function*” column, only the function related with the corresponding category is indicated. Bold type indicates evidences reported in neurons; due to their main importance in rough ER and/or non-neuronal cells, several ER-related functional categories have been omitted, including lipid and glucose synthesis, ER-Golgi interaction and communication, autophagosomes formation, and UPR.*

## Roles of Endoplasmic Reticulum

### ER and Lipid Metabolism

**FIGURE 2:**
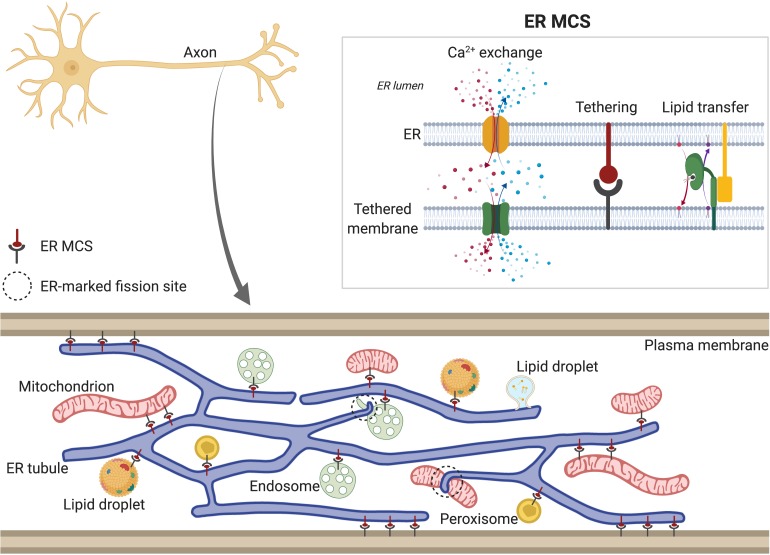
Tubular ER membrane contact sites (MCSs). Tubular ER membrane forms contacts with other membranes in the cell, including plasma membrane (PM), Golgi, mitochondria, endosomes, lipid droplets (LDs) and peroxisomes. Axonal ER **(bottom)** also contains MCSs, except with Golgi, due to its absence in axons. ER MCSs can regulate fission of some of the tethered organelles, such as endosomes and mitochondria (dotted circles). ER MCSs **(top right)** are formed by interactions between ER membrane proteins and proteins found in the other cellular membranes (tethering). This keeps both membranes close enough to allow transfer of lipids and Ca^2+^. Depending on the specific channels localized at each MCS (see main text for details), Ca^2+^ can be released from ER lumen to the other compartment (red) or from other compartment to ER lumen (represented in blue). In both scenarios, Ca^2+^ is released locally to the intramembrane space and then taken up by the acceptor compartment. ER MCSs also mediate non-vesicular and bidirectional transport of lipids, which are shuttled between membranes by lipid transfer proteins (green).

The majority of phospholipids, sterols, sphingolipids, and neutral lipids are synthesized in smooth ER and distributed from there to other cellular compartments. Controlled trafficking of these lipids from/to ER is required to maintain the lipid composition of membranes such as mitochondrial or plasma membrane (PM) (Fagone and Jackowski, 2009; Muallem et al., 2017). Two different transport mechanisms mediate the transfer of lipids between ER and other cellular organelles: vesicular transport is dependent on membrane budding and fusion via the vesicular transport machinery, whereas non-vesicular lipid transport is mediated by ER membrane contact sites (MCSs) with other organelles (Figure 2). In addition, biogenesis of lipid droplets (LDs) (for storage of sterols and triglycerides) and peroxisomes (involved in lipid decomposition through β-oxidation) initiates in the ER. LDs form initially in the ER lipid bilayer, and bud from the ER membrane as they grow. Although it is not still clear how sites of LD formation are determined, some of the ER proteins associated with them have been identified (e.g., seipin) (Szymanski et al., 2007; Salo et al., 2016, 2019) or Syntaxin14 (Datta et al., 2019). These and other proteins, which tether MCS between ER and LDs (discussed below), and control fatty-acid-induced droplet growth, may have roles in determining the sites of nascent LDs. Similarly, peroxisome biogenesis also seems to originate at specific ER domains. In yeast, both peroxisomes and LDs form and remain associated with the same ER subdomains, which contain Pex30 protein (Joshi et al., 2016, 2018), suggesting a link between LD and peroxisome biogenesis at the ER membrane. Although the existence of LDs in neurons has been debated, there is some evidence for their presence, including in axons (reviewed in Pennetta and Welte, 2018).

### ER and Glucose Metabolism

Intracellular glucose is phosphorylated to glucose-6-phosphate (G6P) to be used in metabolism and produce ATP. In tissues like liver, G6P is taken up from cytosol into the ER and dephosphorylated there by G6Pase-α to be returned to cytosol when necessary. Deficiency of G6Pase-α causes glycogen storage disease (Chou et al., 2015). Another G6Pase isozyme, G6Pase-β, is required in astrocytes for glucose storage in their ER lumen. Knockdown of G6Pase-β causes reduction not only of glucose accumulation, but also of Ca^2+^ regulation, and ATP production in the ER of astrocytes. It has been proposed that ER may serve as a glucose intracellular highway. G6Pase-β would facilitate accumulation of glucose to the ER lumen, where it would be free to diffuse and be protected from further metabolism, until returning to the cytosol through ER glucose transporters, and being phosphorylated to G6P to enter glycolysis (Müller et al., 2018). However, it has recently been argued that glucose transport through the ER is too slow (around 550–3,700 times lower than glucose consumption) to be quantitatively relevant for nutrient delivery (Dienel, 2019), and so the physiological role of glucose in the ER lumen is not fully resolved.

### ER and Calcium Dynamics

Release and re-uptake of Ca^2+^ to/from the cytosol mediate initiation and termination of many cellular responses to signals. ER is a major calcium store and manages Ca^2+^ homeostasis by either behaving as a local Ca^2+^ sink, or a store that releases Ca^2+^ to other cellular compartments (Raffaello et al., 2016), controlling processes such as mitochondrial homeostasis and function (Britti et al., 2018). ER can buffer excess presynaptic cytosolic Ca^2+^ during repetitive firing, by uptake via the SERCA calcium ATPase (Sanyal et al., 2005) or potentially also the SPoCk ATPase (Southall et al., 2006). Conversely it can also supply Ca^2+^ to the cytosol via channels including inositol tris-phosphate receptors (IP3R) or ryanodine receptors (RyR) (reviewed by Ross, 2012). A specific role of ER Ca^2+^ has been recently shown in mammalian neurons, where at presynaptic terminals, ER luminal Ca^2+^ can promote PM Ca^2+^ uptake via the ER Ca^2+^ sensor STIM1, a process required for presynaptic Ca^2+^ flux and exocytosis (de Juan-Sanz et al., 2017). Depletion of either the vesicle-associated membrane protein-associated proteins A (VAPA) and B (VAPB), which are ER membrane receptors, or the cytoplasmic VAP-associated protein Secernin-1 (SCRN1), reduces presynaptic Ca^2+^ influx and synaptic vesicle cycling (Lindhout et al., 2019). Also, in *Drosophila* neurons, the ER-resident Ca^2+^ sensor MCTP (multiple C2 domain and transmembrane region protein) promotes release of synaptic vesicles (Genç et al., 2017). Therefore, maintenance of ER Ca^2+^ appears to be crucial for proper synaptic function.

A continuous ER network can also support regional or long-distance Ca^2+^ signaling or homeostasis. Ca^2+^ signals can propagate through the cytosol by Ca^2+^-induced Ca^2+^ release from ER, and thus mediate regional and/or global communication within the cell, analogous to but slower than action potential propagation in the PM. Ca^2+^-induced Ca^2+^ release can be mediated by IP3R or RyR receptors, and be potentiated by elevated cytosolic Ca^2+^ (Straub et al., 2000; Ross, 2012). We know little of the occurrence or roles of propagating Ca^2+^ waves in axons, but a few cases are known. For example, a propagating elevation of cytosolic Ca^2+^ is seen after axonal injury in the early stages of Wallerian degeneration (Vargas et al., 2015). A back-propagating Ca^2+^ wave, which depends on ER Ca^2+^ stores, is also required for the regenerative response to axon injury in dorsal root ganglion (DRG) neurons (Cho et al., 2013). Long-range Ca^2+^ waves also play a role in inhibitory signaling among outgrowing neurites to ensure that only a single neurite will form an axon, although a role for ER in this has not been shown (Takano et al., 2017). All these are situations in which a local event must be communicated to induce responses in other parts of the cell or axon, and where ER continuity can potentially underpin this communication.

The ER lumen can also act as an intracellular highway for Ca^2+^, allowing “Ca^2+^ tunneling”. When luminal Ca^2+^ is released to the cytosol, it must be replenished. The fastest route for replenishment across significant intracellular distances is diffusion through the ER lumen, where there is relatively little Ca^2+^ buffering, leaving Ca^2+^ free to diffuse throughout the lumen of the ER network. This has been shown in non-neuronal cells, including pancreatic acinar cells, *Xenopus* oocytes (reviewed in Petersen et al., 2017) and HeLa cells (Courjaret et al., 2018), but has not been investigated in neurons.

## Axonal ER

Presynaptic terminals can lie up to 1 m from the cell body in human neurons. How can axons mediate communication, and be physically maintained, across this distance? Action potentials at the PM carry long-range signals, and the microtubule (MT) network transports physical cargoes (Hirokawa and Takemura, 2005). A third potential channel for communication along axons is ER, which appears physically continuous throughout neurons (Tsukita and Ishikawa, 1976; Terasaki et al., 1994; Wu et al., 2017; Yalçın et al., 2017) (Figure 1), and has therefore been termed a “neuron within a neuron” (Berridge, 1998, 2002).

An important role for tubular ER is also implied by the genetics of some neurological disorders (Table 2). For instance, mutations in proteins that regulate tubular ER organization are causative for hereditary spastic paraplegia (HSP) and other axonopathies (Hübner and Kurth, 2014; Liberski and Blackstone, 2017). Gradual accumulation of abnormally clustered tubular ER is also found in areas surrounding amyloid plaques in Alzheimer’s disease (AD) brains (Sharoar et al., 2016). Mutation of proteins associated with membrane contacts between ER and mitochondria can also cause diverse neurological defects, including AD, amyotrophic lateral sclerosis (ALS), Parkinson’s disease (PD) or Charcot-Marie-Tooth disease (CMT) (Bernard-Marissal et al., 2018). To understand the impact of axonal ER in neurodegeneration, it is first essential to understand how its organization and dynamics are regulated, and the consequences of disrupting these processes.

**TABLE 2:** Tubular ER-related proteins associated with neurological disorders.

***Protein***	***Roles on/from tubular ER***	***Reported phenotypes in axons***	***Neurological disorders associated***
ARL6IP1	Tubular ER morphogenesis	KD: ER fragmentation and disrupted mitochondrial network organization at presynaptic terminals (Fowler and O’Sullivan, 2016)	**HSP** (Novarino et al., 2014; Wakil et al., 2019)
Ataxin-2	Tubular ER morphogenesis	KD: short and bulged neurites (del Castillo et al., 2019)	**ALS** (Elden et al., 2010) **SCA** (Pulst et al., 1996)
ATL1	Tubular ER morphogenesis LDs growth regulation	KD: Reduced axon growth (Zhu et al., 2006) *KD: Decreased spontaneous release; impaired anterograde transport (De Gregorio et al., 2017); impaired regeneration (Levitan et al., 2016; Rao et al., 2016) *LOF: Fragmented presynaptic ER; Decreased evoked transmitter release (Summerville et al., 2016); increased number of presynaptic terminals (Lee et al., 2009) *OE: Decreased spontaneous release; reduced anterograde transport (De Gregorio et al., 2017); Decreased evoked transmitter release (Summerville et al., 2016)	**HSN** (Guelly et al., 2011) **HSP** (Zhao et al., 2001)
ATL3	Tubular ER morphogenesis Autophagy-mediated tubular ER turnover	LOF: Decreased mitochondrial number (Krols et al., 2019) LOF: Impaired neurite outgrowth (Behrendt et al., 2019) *KD: Decreased spontaneous release; reduced anterograde transport (De Gregorio et al., 2017); impaired regeneration (Levitan et al., 2016; Rao et al., 2016) *LOF: Fragmented presynaptic ER; Decreased evoked transmitter release (Summerville et al., 2016); increased number of presynaptic terminals (Lee et al., 2009) *OE: Decreased spontaneous release; reduced anterograde transport (De Gregorio et al., 2017); Decreased evoked transmitter release (Summerville et al., 2016)	**HSN** (Fischer et al., 2014; Kornak et al., 2014)
BNIP1	Tubular ER morphogenesis ER-LDs tethering		**ALS** (Karch et al., 2018)
INPP5K	Tubular ER morphogenesis	OE: Enhanced neurite outgrowth (Fink et al., 2017)	**Cognitive impairment** (Wiessner et al., 2017)
IP3R	ER-Mitochondria tethering ER-Ca^2+^ transfer to mitochondria Ca^2+^ release	LOF/KD: decreased axon degeneration (Villegas et al., 2014; Orem et al., 2017)	AD (Cheung et al., 2008) **Gillespie syndrome** (Gerber et al., 2016) HD (Tang et al., 2003) HIV-associated sensory neuropathy (Höke et al., 2009) **SCA** (Van De Leemput et al., 2007)
JPH1	ER-PM tethering		**CMT** (Pla-Martín et al., 2015)
JPH3	ER-PM tethering		**HD-like 2** (Holmes et al., 2001)
Lunapark	Tubular ER morphogenesis		**Neurodevelopmental disorder with epilepsy and corpus callosum hypoplasia** (Breuss et al., 2018)
MCTP2	Tubular ER morphogenesis LDs and peroxisomes biogenesis	LOF: Decreased presynaptic release probability and extracellular [Ca^2+^]-sensitive expression of presynaptic homeostatic plasticity (Genç et al., 2017)	**Bipolar disorder** (Djurovic et al., 2009; Scott et al., 2009) **PD** (Latourelle et al., 2009)
MFN2	Tubular ER morphogenesis ER-Mitochondria tethering	LOF: impaired both anterograde and retrograde mitochondrial transport (Misko et al., 2010); synaptic loss (Wang H. et al., 2016); axon degeneration and presynaptic mitochondrial clustering (Zhou et al., 2019)	**CMT** (Züchner et al., 2004) PD (Pham et al., 2012) AD (Wang et al., 2009)
NAG	ER-LDs tethering		**Optic nerve atrophy** (Maksimova et al., 2010)
p600	MT- and ER-interacting protein	KD: impaired neurite extension and decreased ER accumulation at the leading edge of migrating axons (Shim et al., 2008)	**Episodic ataxia** (Conroy et al., 2014) PD (Zhang et al., 2010b)
PTP1B	Dephosphorylation of the endosome protein EGFR		AD (Pei et al., 1994; Vieira et al., 2017)
Rab10	Tubular ER morphogenesis ER movement along MTs	KD: Impaired trafficking of membrane precursor vesicles during axon growth (Wang et al., 2011; Deng et al., 2014; Xu et al., 2014) KD/LOF/OE: impaired dense core vesicles secretion (Sasidharan et al., 2012)	AD (Ridge et al., 2018)
Rab18	Tubular ER morphogenesis ER-LDs tethering	KD: impaired neurite outgrowth (Wu Q. et al., 2016) LOF: axon degeneration (Cheng et al., 2015); disorganized cytoskeleton and accumulation of MTs at the presynaptic terminals (Carpanini et al., 2014)	**WARBM** (Bem et al., 2011)
Rab3GAP1	Tubular ER morphogenesis ER-LDs tethering	LOF: inhibited Ca^2+^-dependent neurotransmitter release (Sakane et al., 2006); blocked synaptic homeostatic response (Müller et al., 2011)	**WARBM** (Aligianis et al., 2005)
Rab3GAP2	Tubular ER morphogenesis ER-LDs tethering	KD: impaired neurite outgrowth (Wu Q. et al., 2016)	**HSP** (Novarino et al., 2014) Martsolf syndrome (Aligianis et al., 2006) **WARBM** (Borck et al., 2011)
REEP1	Tubular ER morphogenesis ER-Mitochondria tethering ER-MTs interaction LDs growth regulation	LOF: impaired neurite outgrowth (Lim et al., 2015); axon degeneration (Beetz et al., 2013); large lysosomes (Allison et al., 2017) OE: neuritic degeneration (Lim et al., 2015) *LOF: partial loss of ER at distal regions (Yalçın et al., 2017)	**HSP** (Züchner et al., 2006) **Spinal CMT** (Beetz et al., 2012)
REEP2	Tubular ER morphogenesis	*LOF: partial loss of ER at distal regions (Yalçın et al., 2017)	AD (Sharoar et al., 2016) **HSP** (Esteves et al., 2014)
REEP5	Tubular ER morphogenesis	*LOF: partial loss of ER at distal regions (Yalçın et al., 2017)	AD (Sharoar et al., 2016)
RTN2	Tubular ER morphogenesis	*KD: partial loss of ER and MTs at distal regions; increased size and reduced number of mitochondria at presynaptic terminals (O’Sullivan et al., 2012) *LOF: partial loss of ER and occasional network discontinuities at distal regions; impaired anterograde transport (Yalçın et al., 2017)	**HSP** (Montenegro et al., 2012)
RTN3	Tubular ER morphogenesis Autophagy-mediated tubular ER turnover	OE: reduced anterograde transport (Shi et al., 2009; Deng et al., 2013); ER clustering; reduced size and increased number of mitochondria (Sharoar et al., 2016) *KD: partial loss of ER and MTs at distal regions; increased size and reduced number of mitochondria at presynaptic terminals (O’Sullivan et al., 2012) *LOF: partial loss of ER and occasional network discontinuities at distal regions; impaired anterograde transport (Yalçın et al., 2017)	AD (Sharoar et al., 2016) Diabetes-induced neuropathy (Zhao et al., 2013)
RTN4	Tubular ER morphogenesis	LOF/KD: enhanced regeneration (associated with the inhibition of RTN4 in oligodendrocytes) (Chen et al., 2000; GrandPré et al., 2000; Vajda et al., 2015) LOF: reduced regeneration (associated with inhibition of RTN4 in neurons) (Vajda et al., 2015) *KD: partial loss of ER and MTs at distal regions; increased size and reduced number of mitochondria at presynaptic terminals (O’Sullivan et al., 2012) *LOF: partial loss of ER and occasional network discontinuities at distal regions; impaired anterograde transport (Yalçın et al., 2017)	ALS (Dupuis et al., 2002) Multiple sclerosis (Karnezis et al., 2004)
RyR	ER-Ca^2+^ release	KD: decreased axon degeneration (Villegas et al., 2014); decreased synaptic facilitation (Zhang et al., 2009; Wu et al., 2013); decreased presynaptic Ca^2+^ (Shimizu et al., 2008)	AD (Del Prete et al., 2014)
Sec22b	ER-PM tethering Interaction with lipid transfer proteins	LOF: impaired axon growth (Petkovic et al., 2014)	AD (Zhao et al., 2016)
Sec61β	ER-MTs interaction		PD (Wallen et al., 2018)
Seipin	ER-LDs tethering LDs growth regulation	KD: impaired regeneration (Rao et al., 2016)	**CMT** (Auer-Grumbach et al., 2005) **HSP** (Windpassinger et al., 2004) PD (Licker et al., 2014) **Silver syndrome** (Windpassinger et al., 2004) **Spinal CMT** (Windpassinger et al., 2004) **Progressive encephalopathy** (Guillén-Navarro et al., 2013)
SERCA	ER-Ca^2+^ uptake	KD: decreased presynaptic Ca^2+^ and exocytosis (de Juan-Sanz et al., 2017); decreased amplitude of excitatory postsynaptic potentials (Desai-Shah and Cooper, 2009)	AD (Krajnak and Dahl, 2018) Diabetes-induced neuropathy (Verkhratsky and Fernyhough, 2008) PD (Lee J.H. et al., 2019)
SigR1	Regulation of ER-Ca^2+^ release channels	KD: decreased axonal length (Tsai et al., 2015) LOF: impaired anterograde transport (Tsai et al., 2015)	AD (Mishina et al., 2008; Hedskog et al., 2013) **ALS** (Al-Saif et al., 2011; Prause et al., 2013) Chemotherapy-induced peripheral neuropathy (Bruna and Velasco, 2018) HD (Hyrskyluoto et al., 2013; Miki et al., 2015; Ryskamp et al., 2017) PD (Mishina et al., 2005; Mori et al., 2012) **Spinal CMT** (Li et al., 2015)
spastin	ER-Endosomes tethering MT severing LDs growth regulation	KD: impaired axon outgrowth (Wood et al., 2006); reduced presynaptic area, increased excitatory junction potential amplitude, and accumulation of stabilized MTs (Trotta et al., 2004); loss of LDs (Papadopoulos et al., 2015); enhanced endosomal tubulation (Allison et al., 2013) LOF: reduced axonal length (Solowska et al., 2008); impaired regeneration (Stone et al., 2012); decreased presynaptic terminals size, decreased quantal content, and presynaptic loss of stabile MTs (Sherwood et al., 2004); large lysosomes (Allison et al., 2017) OE: reduced excitatory junction potential amplitude and presynaptic loss of stabile MTs (Trotta et al., 2004); increased LDs size and number (Papadopoulos et al., 2015)	**HSP** (Hazan et al., 1999)
STIM1	ER-PM tethering Regulation of ER-Ca^2+^ uptake ER-MTs interaction	KD: impaired presynaptic Ca^2+^ influx and exocytosis (de Juan-Sanz et al., 2017) LOF/OE: impaired axon guidance (Shim et al., 2013) LOF: reduced ER translocation into growth cone filopodia (Pavez et al., 2019)	AD (Gutierrez-Merino et al., 2018)
STIM2	Regulation of ER-Ca^2+^ uptake		AD (Bojarski et al., 2009; Sun et al., 2014)
Syntaxin14	LDs growth regulation at ER-LDs contacts	LOF: reduced myelinated axonal tracts with vacuolization (Akizu et al., 2015)	**SCA** (Thomas et al., 2014; Shukla et al., 2017)
TBC1D20	LDs growth		**WARBM** (Liegel et al., 2013)
VAPB	Tubular ER morphogenesis Tubular ER dynamics ER MCSs tethering via interaction with lipid transfer proteins	KD: decreased synaptic activity (Gómez-Suaga et al., 2019) *KD: ER discontinuity, impaired ER tubules dynamics, and decreased presynaptic Ca^2+^ influx and synaptic vesicle cycling (Lindhout et al., 2019) *LOF: impaired axonal localization of Dscam cell surface receptors (Yang et al., 2012); decreased number and increased size of presynaptic terminals, and impaired presynaptic MT network (Pennetta et al., 2002); decreased presynaptic BMP signaling (Ratnaparkhi et al., 2008) *OE: increased number and decreased size of presynaptic terminals (Pennetta et al., 2002); decreased presynaptic active zones (Ratnaparkhi et al., 2008)	**ALS** (Nishimura et al., 2004) **SMA** (Nishimura et al., 2004)
VPS13A	ER-Mitochondria tethering ER-LDs tethering	KD: neurite degeneration (Park et al., 2015)	**Chorea-acanthocytosis** (Ueno et al., 2001; Shen et al., 2017)
VPS13C	ER-Endosome/Lysosome tethering ER-LDs tethering		**PD** (Lesage et al., 2016; Schormair et al., 2018)
**^#^**VPS13D	Regulation of mitochondrial size and clearance	KD: decreased mitochondrial content in distal axons (Seong et al., 2018)	**HSP/ataxia** (Gauthier et al., 2018; Seong et al., 2018)

In the “*Protein*” column, **#** indicates a protein potentially related with tubular ER. Detailed information and references for the indicated functions can be found in Table 1 and the main text; in the “*Reported phenotypes in axons*” column, * refers to evidences reported by affecting not only the corresponding specific protein, but several or all the members of the protein family. *KD*, *Knock-down; LOF*, *loss of function; OE*, *overexpression;* in the “*Neurological disorders associated*” column, bold type indicates neurological disorders where mutations or variants in the corresponding gene were found in patients; for other disorders, there is evidence relating the protein with the pathogenic mechanisms, but not necessarily as a primary cause of the disease. *AD*, *Alzheimer’s disease; ALS*, *amyotrophic lateral sclerosis; CMT*, *Charcot-Marie-Tooth disease; HSN*, *hereditary sensory neuropathy; HSP*, *hereditary spastic paraplegia; HD*, *Huntington’s disease; PD*, *Parkinson’s disease; SCA*, *spinocerebellar ataxia; SMA*, *spinal muscular atrophy; WARBM*, *Warburg micro syndrome.*

### Axonal ER Organization

Local ER organization is mediated by a group of evolutionary conserved protein families found in the ER membrane, each one specialized in regulating particular aspects of ER morphology. Axonal ER is mostly composed of tubular ER (Figure 1). Therefore, to understand axonal ER biology, it is critical to know how the tubular ER network is regulated. It is well established that reticulons (RTNs) and REEP proteins drive formation of ER tubules (Voeltz et al., 2006; Beetz et al., 2013). In yeast, these two protein families are together essential for most peripheral ER tubules (Hu et al., 2008), although in other organisms like *Drosophila*, the absence of RTNs and REEPs is not enough to abolish ER tubule formation in axons (Yalçın et al., 2017), indicating that additional proteins are involved in formation of ER tubules in neurons. RTNs and REEPs are proposed to control ER tubulation through their hydrophobic intramembrane domains, which insert in the cytosolic face of the ER lipid bilayer, distorting it and inducing local curvature of the bilayer (Shibata et al., 2009) (Figure 1). Also, Pex30, a yeast protein enriched at sites of nascent LDs and peroxisomes on ER, is found in tubules and the edges of sheets, as are RTNs. Pex30 has a reticulon homology domain (RHD), as does its closest human homolog, MCTP2. Expression of Pex30 (Joshi et al., 2016) or MCTP2 RHD restores ER tubules in yeast lacking RTNs (Joshi et al., 2018). Therefore, in addition to their role in lipid metabolism, Pex30/MCTP2 may have a redundant role with RTNs and REEPs in tubular ER formation.

Other proteins that can shape tubular ER include ADP-ribosylation factor-like 6 interacting protein 1 (ARL6IP1) (Yamamoto et al., 2014) and protrudin (Chang et al., 2013). Both are found in tubular ER and possess hairpin-loop domains like RTNs and REEPs. However, the presence of a short polar stretch in the middle of their potential hairpins suggest that they span the entire ER membrane. Nevertheless, there is evidence for roles for both ARL6IP1 and protrudin in shaping tubular ER. In *Drosophila* larval motor neurons, ARL6IP1 knockdown disrupts ER distribution at axon terminals (Fowler and O’Sullivan, 2016). In addition, ARL6IP1 recruits INPP5K, an inositol 5-phosphatase enriched in newly formed ER tubules. Loss of ARL6IP1 or INPP5K leads to an increase in ER sheets (Dong et al., 2018), consistent with the idea that ARL6IP1 promotes formation of ER tubules. Also, protrudin depletion in mammalian cells alters ER network morphology, promoting the extension of ER sheets (Chang et al., 2013), again consistent with a role in promoting formation of ER tubules.

Axonal ER forms a network of interconnected tubular structures. Fusion between ER tubules is mediated by the atlastins (ATLs), a family of GTPases that mediate fusion between ER membranes in a GTP-dependent manner (Orso et al., 2009). As in yeast and mammalian cell culture models (Hu et al., 2009; Kornak et al., 2014; Wang S. et al., 2016), depletion of ATL in *Drosophila* neurons leads to tubular ER fragmentation and unbranched ER tubules (Orso et al., 2009). Similar branching defects have also been reported in *C. elegans* neurons mutated in *atln-1*, the *ATL1 ortholog* (Liu et al., 2019). In addition to their GTPase catalytic domain, ATLs also possess intramembrane domains, similar to those found in RTNs and REEPs, that are not only essential for ATL membrane fusion activity, but also could explain why ATL1 drives the generation of membrane tubules from proteoliposomes *in vitro* (Betancourt-Solis et al., 2018).

Another protein with an ER intramembrane domain is the M1 form of the MT-severing protein spastin (Park et al., 2010), which interacts with RTNs (Mannan et al., 2006), REEPs and ATLs (Park et al., 2010). However, there is not yet any evidence for a direct role for spastin in promoting formation of ER tubules. Similarly, a potential role of FAM134B protein in ER tubulation is not clear. FAM134B contains a predicted RHD, and its loss produces expanded ER sheets. Also, via its RHD, FAM134B binds to liposomes and produces highly curved proteoliposomes. However, FAM134B is mainly found in perinuclear ER, where it has been proposed to promote curvature at the edges of sheets (Khaminets et al., 2015; Bhaskara et al., 2019).

ER-shaping proteins that mediate tubulation, such as RTNs and REEPs, localize not only in tubular ER, but also at the edges of ER sheets (Shibata et al., 2010), both regions with high curvature. Another ER-resident protein whose localization is related to its function is Lunapark. This protein is directly involved in shaping the ER tubular network by stabilizing three-way junctions between ER tubules, where it also localizes (Chen et al., 2012, 2015). Absence of Lunapark in mammalian cells increases the amount of ER sheets, but does not abolish the formation of an ER tubular network (Wang S. et al., 2016; Zhou et al., 2018), suggesting that although required to stabilize the three-way junctions, Lunapark is not essential to develop ER tubules.

Roles in shaping tubular ER are not confined to proteins with intramembrane hairpin domains. For example, the small GTPases Rab10 and Rab18 regulate tubular ER morphology: depletion of Rab10 produces expansion of cisternal ER and fewer ER tubules (English and Voeltz, 2013), and loss of Rab18 from ER tubules, by depletion of the Rab3GAP complex (which is also a Rab18 GEF), causes fragmentation of the ER tubular network and spread of ER sheets (Gerondopoulos et al., 2014). Another example is the RNA-binding protein Ataxin-2, also associated with ER, whose depletion causes formation of ER aggregates in the peripheral ER network of *Drosophila* neurons (del Castillo et al., 2019). Mitofusin-2 (MFN2) is found at both mitochondria and ER membranes, being particularly enriched at the latter. A role for ER MFN2 in ER tubulation has been proposed, since it is required to keep luminal continuity at the peripheral ER in mammalian cells (de Brito and Scorrano, 2008). VAPA/B are also found at ER membrane and required for axonal ER continuity (Lindhout et al., 2019).

Finally, one characteristic feature of axonal ER is its very small diameter – ER tubules in most cells frequently has a diameter of ∼60 nm (varying between 25 and 90 nm), but in axons, ER tubules have a diameter on average around 40 nm, and often becoming small enough for the lumen not to be even visible (Wu et al., 2017; Yalçın et al., 2017; Terasaki, 2018). The reasons for this specialization are unclear, but the predicted consequences include limited capacity to either release or sequester Ca^2+^, as well as limitations on both Ca^2+^ tunneling and glucose tunneling. Therefore, while ER continuity appears to be important for its function, there also appear to be reasons to constrain lumen continuity.

### Axonal ER Membrane Contact Sites (MCSs)

The continuity of the axonal ER network suggests a role for it in communicating throughout neurons, but also provides capacity to regulate cellular processes at local or regional levels. Tubular ER has MCSs with nearly every membranous organelle, including PM (Elbaz and Schuldiner, 2011; Phillips and Voeltz, 2016; Wu et al., 2018) (Figure 2). This is also true for axonal ER, where focused ion-beam scanning electron microscopy (FIB-SEM) reveals membrane domains in close proximity to other organelles (Wu et al., 2017). Although most of our understanding of ER MCSs comes from yeast and mammalian non-neuronal cells (Eisenberg-Bord et al., 2016), the molecular machinery associated with these MCSs is present in axons (Bernard-Marissal et al., 2018). ER MCSs are not mere physical attachments between ER and other organelles; depending on both the organelle and their function, they can be enriched for different proteins. For example, some MCSs are related to Ca^2+^ exchange, others for lipid exchange (Eisenberg-Bord et al., 2016). Impairment of these MCSs disrupts organellar communication and can cause diverse cellular defects, including in organelle dynamics (Friedman et al., 2011; Mandikian et al., 2014; Rowland et al., 2014), lipid metabolism (Bian et al., 2017; Hua et al., 2017) or Ca^2+^ levels (Bernard-Marissal et al., 2015; de Juan-Sanz et al., 2017). Here, we summarize the different MCS known between ER and other organelles, and those MCSs reported in neurons.

#### ER-Mitochondria MCS

In mouse neurons, axonal ER tubules typically contact around 5% of mitochondrial surface (Wu et al., 2017). Although it is still unknown which signals control the formation and maintenance of these MCSs, we know about some molecules located there. ER-shaping protein REEP1 is reported not only at ER membrane, but also at mitochondrial membrane, and to be required for ER-mitochondria interactions in non-neuronal cells (Lim et al., 2015). A role for MFNs in ER-mitochondria tethering has been also reported in non-neuronal cells (de Brito and Scorrano, 2008), and they are proposed to have a similar role in neurons, where they are required for proper mitochondrial dynamics (Züchner et al., 2004). Another tethering complex in neuronal cells comprises VAPB (at ER membrane) bound to the mitochondrial protein tyrosine phosphatase-interacting protein 51 (PTPIP51) (De Vos et al., 2012; Stoica et al., 2014). The VAPB-PTPIP51 tether has been specifically reported in presynaptic regions; neuronal activity increases presynaptic VAPB-PTPIP51 MCSs; and loss of either VAPB or PTPIP51 reduces synaptic activity, suggesting an important role for VAPB-PTPIP51 MCSs in synaptic function (Gómez-Suaga et al., 2019). Also in neurons, the ER membrane protein PDZD8 promotes ER-mitochondria MCSs (Hirabayashi et al., 2017).

ER-mitochondria MCSs are required for diverse processes, including controlling mitochondrial Ca^2+^ levels. ER is a major Ca^2+^ source, and mitochondria can take up Ca^2+^ from ER via ER-mitochondria MCSs, where Ca^2+^ exits the ER lumen through the IP3R, which is required for maintaining ER-mitochondria MCSs (Bartok et al., 2019). IP3R is stabilized by the ER chaperone Sigma receptor-1 (SigR1) (Hayashi and Su, 2007), and binds to the voltage-dependent anion channel (VDAC) on mitochondrial outer membrane (Cárdenas et al., 2010). IP3R-VDAC interaction is stabilized by the molecular chaperone glucose-regulated protein 75 (grp75) (Szabadkai et al., 2006). After exiting the ER lumen, Ca^2+^ is translocated into mitochondria by calcium uniporters. Mitochondrial Ca^2+^ levels control ATP production by activation of key metabolic enzymes in the Krebs cycle (Gellerich et al., 2010), and at high levels, promote apoptosis by opening the mitochondrial permeability transition pore (mPTP) and allowing release of mitochondrial contents to the cytosol (Baumgartner et al., 2009). Therefore, ER-mitochondria MCSs have a critical role in mitochondrial function by controlling mitochondrial Ca^2+^ levels. Enrichment and function of VDAC, IP3R, SigR1 or grp75 proteins at ER-mitochondria MCSs has been specifically reported in neurons (Shoshan-Barmatz et al., 2004; Higo et al., 2010; Mavlyutov et al., 2010), including in axons (Bernard-Marissal et al., 2015).

Some of the enzymes required for the biosynthesis of mitochondrial lipids, such as phospholipids or cholesterol, are found in the ER membrane, and interestingly, enriched at ER-mitochondria MCSs, where lipids are exchanged between both organelles (Vance, 2014). Mitochondrial function (Tasseva et al., 2013) and balanced fusion and fission (Steenbergen et al., 2005) are dependent on specific lipid composition, and therefore, lipid exchange at ER-mitochondria MCSs may be critical in regulating these processes. The Vps13 family of lipid-transfer proteins are localized to a variety of intracellular membrane contacts; of these, Vps13A (vacuolar protein sorting-associated protein 13A), which directly interacts with VAPA (Yeshaw et al., 2019), is found at mammalian ER-mitochondrial MCSs (Kumar N. et al., 2018), although it is not known how much it contributes to lipid transport there (Petrungaro and Kornmann, 2019). Oxysterol binding protein (Osbp)-related proteins ORP5 and ORP8 are also found at ER-mitochondria MCS, where they mediate non-vesicular transport of phosphatidylserine from the ER to mitochondria in mammalian cells (Rochin et al., 2019).

Mitochondrial fusion and fission events are driven by membrane-bound GTPases like MFNs and dynamin related protein (DRP-1) (Hoppins et al., 2007). In addition to the above-mentioned role for MFNs in tethering ER-mitochondria MCSs, DRP-1 recruitment to mitochondria occurs after ER tubules mark the mitochondrial constriction site (Friedman et al., 2011), suggesting a critical role for ER-mitochondria MCSs in mitochondrial dynamics. Moreover, a role for tubular ER in this process is supported by the fact that knockdown of key ER-shaping proteins such as ATL3, RTN1, RTN4, or Lunapark produces structural alteration of mitochondria in human cells (Milani et al., 2018; Krols et al., 2019). These evidence comes from non-neuronal cells, but since ER-shaping proteins control tubular ER in axons (see above), it is reasonable to expect that axonal ER regulates mitochondrial dynamics similarly. Another role attributed to ER-mitochondria MCSs and pending exploration in neurons, is the formation of autophagosomes (Hamasaki et al., 2013), which mediate the fundamental cellular process of autophagy (Mizushima et al., 2011).

#### ER-PM MCS

Contacts between large flat ER cisternae and PM are mostly seen in neuronal cell bodies, but also in axons, where between 0.5 and 1% of the PM surface contacts ER (Wu et al., 2017). Depending on function, these MCSs can be mediated by various ER membrane proteins, allowing exchange of particular lipids or Ca^2+^ between ER and PM membranes (Saheki and Camilli, 2017).

Ca^2+^ exchange at ER-PM MCSs can be mediated by different molecular complexes. In non-neuronal cells, the ER Ca^2+^ sensor STIM1 binds a store-operated channel (SOC), the PM Ca^2+^ channel Orai1, in a process stimulated by Ca^2+^ depletion in ER to induce Ca^2+^ influx from the extracellular medium (Carrasco and Meyer, 2011). In contrast to its stabilizing role on the ER-Ca^2+^ release channel IP3R (see above), SigR1 associates with STIM1 and attenuates its interaction with Orai1 (Srivats et al., 2016). In neurons, the role described for STIM1 seems to be mostly performed by its paralog STIM2 (Berna-Erro et al., 2009), whereas STIM1 activation in presynaptic terminals locally modulates presynaptic function, impacting activity-driven Ca^2+^ entry and release probability (de Juan-Sanz et al., 2017).

PM voltage-gated K^+^ channels (Kv) and ER RyR Ca^2+^ channels also accumulate at ER-PM MCSs in neurons (Mandikian et al., 2014), controlling local Ca^2+^ homeostasis and the formation of these MCSs (Fox et al., 2015; Kirmiz et al., 2018). Other molecules related to ER-PM MCSs are junctophilins (JPHs), ER membrane proteins that bind the PM through their N-terminal domain. In addition to tethering both membranes, JPHs have a role in PM-ER Ca^2+^ signaling, potentially by their interaction with Ca^2+^ channels and/or facilitating the interaction between Ca^2+^ channels from both membranes (Landstrom et al., 2014). Although this role has been mainly studied in muscle junctional membrane complexes, which are formed between the sarcoplasmic reticulum and T-tubule membranes, there is evidence supporting a similar role for JPHs in neurons (Moriguchi et al., 2006; Sahu et al., 2019).

ER-PM MCSs also allow transport of lipids. Similar to mitochondria, enzymes for the biosynthesis of some PM lipids are found in ER membranes. This compartmentalization of lipid metabolic enzymes facilitates regulation of the lipid composition of both membranes (Lauwers et al., 2016), which affects key cellular processes such as the actin cytoskeleton, membrane protein recruitment, and vesicle trafficking (Toker, 2002). Extended synaptotagmins (E-Syts), integral ER membrane proteins, bind to PM phosphatidyl inositol 4,5 bisphosphate (PI(4,5)P_2_) and transfer glycerophospholipids between ER and PM in a Ca^2+^-dependent manner (Yu et al., 2016; Bian et al., 2017) harboring lipids within their SMP (synaptotagmins-like-mitochondrial-lipid binding protein) domain (Schauder et al., 2014; Jeong et al., 2017). In *Drosophila* neurons, *E*-Syt localizes at axonal ER, and is required for proper neurotransmission and synaptic growth. However, at least in the presynaptic region, E-Syt may not be required for lipid homeostasis, since synaptic balance of phospholipids PI(4,5)P_2_ and PI(3)P appears normal in E-Syt mutants (Kikuma et al., 2017). Transmembrane protein 24 (TMEM24), also regulated by Ca^2+^ and with SMP domains, is enriched at ER-PM MCSs, where it acts as a tether and transfers phospholipids in mammalian neurons, including in axons (Sun et al., 2019).

As at MCSs between ER and other organelles, VAP proteins mediate tethering between ER and PM membranes, indicating a general role for these proteins in the formation of ER MCS. VAPs can recruit diverse proteins with lipid-binding domains to the ER by binding to FFAT [diphenylalanine (FF) in an acidic tract] motifs in these proteins, allowing exchange of phospholipids, ceramides or sterols between both membranes (Murphy and Levine, 2016). Moreover, the SNARE proteins Sec22b (ER-resident) and Syntaxin1 (PM protein) mediate non-fusogenic MCSs between ER and PM in neurons. This tether contributes to PM expansion in growth cones (Petkovic et al., 2014), a process thought to be controlled by non-vesicular lipid transfer (Gupton and Gertler, 2010). Since Sec22 interacts with lipid transfer proteins Osh2 and Osh3 (oxysterol-binding homology proteins 2 and 3; phosphoinositide-binding proteins) in yeast, it is proposed that Sec22b-Syntaxin1 interaction may regulate neuronal PM growth by controlling lipid synthesis and transfer (Petkovic et al., 2014).

#### ER MCS and Intracellular Membrane Trafficking

Endocytosed molecules from the PM are delivered to endosomes, where they are sorted for traffic to other compartments, for example to lysosomes for degradation (Elkin et al., 2016). ER and endosomes form dynamic MCSs, where both organelles are linked to MTs (Friedman et al., 2013). ER-endosome MCSs are reported in axons, where ER contacts tubulovesicular structures (early endosomes), multivesicular bodies (late endosomes) and lysosomes (Wu et al., 2017). MCSs between ER and endosomes can be mediated by VAP and lipid-binding proteins, allowing exchange of lipids like cholesterol or phospholipids (Eden, 2016). One lipid-transfer protein associated with these MCSs is Vps13C (Kumar N. et al., 2018). In addition, endosomal cargoes can interact with ER membrane proteins, as revealed by the interaction between the ER-localized protein tyrosine phosphatase PTP1B and the endosome-associated epidermal growth factor receptor (EGFR). In this interaction, PTP1B dephosphorylates EGFR and leads its degradation (Eden et al., 2010). Similar than its role on mitochondrial dynamics, ER contacts define the position of endosome fission (Rowland et al., 2014; Hoyer et al., 2018). No evidence of lipid transfer between ER and endosomes have been reported in axons yet. However, a role for tubular ER (including in neurons) in regulating endosomal dynamics has been proposed. The ER-shaping protein and MT-severing ATPase spastin, is required to initiate endosomal tubule fission at ER-endosomal MCSs. Failure in this process causes increased endosomal tubulation (Allison et al., 2013) and abnormal lysosomal morphology (Allison et al., 2017).

Both LDs and peroxisomes, with roles in lipid storage and lipid degradation respectively, can be generated from ER (Joshi et al., 2018). Moreover, the distribution and function of these two organelles depend on their contacts with ER (Ohsaki et al., 2017; Farré et al., 2018). ER-peroxisome contacts can be mediated by VAP proteins and the endosomal-associated protein acyl-coenzyme A–binding domain protein 5 (ACBD5) (Costello et al., 2017; Hua et al., 2017). In neurons, overexpression of ACBD5 produces diminished peroxisomal long-range movements, along with accumulation of peroxisomes in dendrites and axons (Wang Y. et al., 2018).

During LD biogenesis, membrane continuity is observed between LD and ER, forming membrane bridges. In addition, LDs also associate with the ER, at sites where protein complexes tether the membranes of both organelles. Molecules identified at these tethers include a complex formed by diacylglycerol *O*-acyltransferase 2 (DGAT2) (in LD membrane) and the fatty acid transport protein FATP1 (in ER membrane) (Xu et al., 2012); one formed by LD-associated Rab18 with the ER-associated proteins NAG-RINT1-ZW10 (NRZ complex) and Syntaxin18, Use1 and BNIP1 (SNARE proteins) (Xu et al., 2018); and tethering mediated by Vps13A and Vps13C (Kumar N. et al., 2018). At least one of them, Vps13A, interacts with the ER-membrane protein VAP-A (Yeshaw et al., 2019). These tethers, which are involved in LD biogenesis, have not been reported in neurons yet. Nevertheless, ER membrane proteins REEP1 (Falk et al., 2014) and spastin (Papadopoulos et al., 2015) are associated with LDs in neurons, controlling LD distribution and size. A similar role has been reported in non-neuronal cells for additional ER membrane proteins, such as ATL (Klemm et al., 2013), seipin (Wang H. et al., 2016; Salo et al., 2019), LD-associated hydrolase (LDAH) (Goo et al., 2017) or Syntaxin14 (Datta et al., 2019). Interestingly, ATL (Zhu et al., 2003), seipin (Ito et al., 2008) and LD-associated hydrolase (LDAH) *Drosophila* ortholog (Thiel et al., 2013; Yalçın et al., 2017), localize at neuronal ER tubules. Therefore, they may perform a similar role in LD biogenesis in neurons.

In cell bodies and in non-neuronal cells, ER exchanges material with the Golgi apparatus via both vesicle trafficking, and non-vesicular transport mediated by MCSs (Sassano and Agostinis, 2019). These contacts between ER and Golgi also depend on VAP and lipid-binding proteins, allowing exchange of lipids such as ceramides or phospholipids. Although canonical Golgi structures have not been observed outside the cell body, Golgi-derived structures, called Golgi outposts, are found in dendrites (Gardiol et al., 1999; Pierce et al., 2001; Horton and Ehlers, 2003). Golgi outposts have been also reported in axons during synapse development (Sytnyk, 2003) and in mature axons (González et al., 2016), mediating vesicular trafficking with ER, and revealing a machinery for the local processing of membrane proteins. Whether axonal ER and Golgi outposts also possess MCS remains unknown.

### Axonal ER Dynamics

During cell development, ER material is organized and distributed through the cell (Bobinnec et al., 2003). For example, in axon growth cones, dynamic ER tubules drive ER network extension into the cell periphery (Dailey and Louis, 1989; Farías et al., 2019). During these stages, ER tubule distribution is not “sculpted” to remain unalterable, but remains highly dynamic even in differentiated cells and neurons. New tubules grow from old ones, old tubules retract, new junctions appear, and cisternae relocate. Remarkably, this dynamic nature is evolutionary conserved, being observed in plants (Stefano and Brandizzi, 2017), yeast (Du et al., 2004) and animal cells (Lee and Chen, 1988; Nixon-Abell et al., 2016; Schroeder et al., 2018), indicating that the capacity of tubular ER to change its distribution plays a critical role in cell responses to physiological changes [i.e., ER stress (Schuck et al., 2009) or Ca^2+^ levels (Ribeiro et al., 2000; Brough et al., 2005), or to control processes such as cell motility (Hernandez et al., 2006) or cell division (Bobinnec et al., 2003; Lu et al., 2009)]. The continuity of the ER network within the confined dimensions of axons obscures some features of ER dynamics, but anterograde and retrograde transport of ER tubules can be seen superimposed on the apparent stability of the continuous network (Yalçın et al., 2017). Since the axonal ER network is dynamic, since the ER assembly mechanisms appear to be quite efficient at maintaining a continuous network and avoiding gaps even when ER tubule numbers are significantly reduced, and since there appear to be no physical constraints on an even denser network of ER tubules (Yalçın et al., 2017), we suggest the existence of homeostatic mechanisms to achieve the physiological density and continuity of ER tubules. These mechanisms would ensure the presence of enough ER tubules to maintain continuity by fusing and joining up gaps in the network, while avoiding the presence of excess ER tubules; such mechanisms would depend on a pool of free mobile tubules through which excess tubules can be transported away, or required tubules delivered.

#### MT-Mediated Tubular ER Dynamics

**FIGURE 3:**
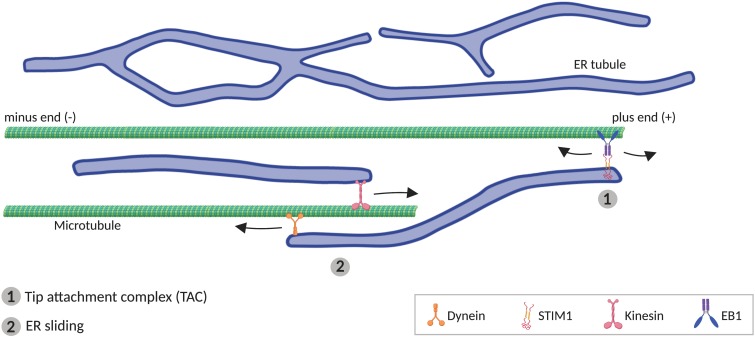
Microtubule (MT)-mediated transport of ER tubules. (1) Tip attachment complex (TAC) is formed between a MT plus end and the tip of an ER tubule through STIM1-EB1 interaction, resulting in movement of the ER tubule when the MT grows or retracts. (2) In ER sliding mechanism, the tip of an ER tubule associates to MTs, where motor proteins kinesin and dynein mediates, respectively, anterograde and retrograde movement of the ER tubule.

Although some components of the ER-shaping machinery are sufficient to generate tubules *in vitro* (i.e., RTNs), MTs are additionally required *in vivo* (Terasaki et al., 1986; Dailey and Louis, 1989; Lu et al., 2009; Farías et al., 2019). In animal cells, peripheral ER tends to co-align with MTs (Terasaki et al., 1986; Kimura et al., 2017; Farías et al., 2019), which drive the movement of ER tubules. Two different mechanisms have been described for how ER tubules move alongside MTs (Figure 3). One of these involves movement of individual ER tubules on extending MTs, mediated by the tip attachment complex (TAC). In TACs, the tip of an ER tubule is attached to the tip of a MT plus end, through a complex containing the ER protein STIM1 and the MT-associated protein EB1. This binding leads to the ER tubule movement when the MT grows or retracts (Grigoriev et al., 2008; Pavez et al., 2019). The other mechanism mediating ER tubule movement along MTs, faster and more frequent than TAC, is ER sliding, driven by MT motor proteins such as kinesin-1 and dynein (Woźniak et al., 2009). In ER sliding, the tip of an ER tubule binds to stable (acetylated) MTs, and ER movements result from ER transport along the MT. Since this movement is MT-motor dependent, it does not necessarily correlate with MT growth or shrinkage (Lee and Chen, 1988; Waterman-Storer and Salmon, 1998; Woźniak et al., 2009). TAC-mediated movement and ER sliding are independent of each other, for example, ER sliding is still observed when STIM1 or EB1 are mutated (Grigoriev et al., 2008), or in the presence of nocodazole, which depolymerizes non-acetylated MTs (Terasaki et al., 1986; Waterman-Storer and Salmon, 1998).

STIM2 and EB3, paralogs of STIM1 and EB1 respectively, are preferentially expressed in the central nervous system (Nakagawa et al., 2000; Skibinska-Kijek et al., 2009), and they also physically bind each other (Grigoriev et al., 2008), what suggests that STIM2-EB3 complex may mediates TAC in neurons. However, although knockdown of both EB1 and EB3 reduces dendritic ER expansion, it does not affect axonal ER distribution (Farías et al., 2019), suggesting that TAC mechanism may not be essential for axonal ER transport. Conversely, knockdown of kinesin-1 or dynein disrupts anterograde or retrograde transport, respectively, of ER tubule along the axon (Farías et al., 2019), supporting a critical role for ER sliding in axonal ER transport. In spite of this, it is still not clear which ER molecules serve as adaptors. One component of the complex that binds ER and MTs may be Protein 600 (p600), which appears associated with MTs and ER tubules in neurons (Shim et al., 2008). In addition, several integral ER membrane proteins are able to bind MTs. One of them is kinectin, proposed as an ER adaptor for kinesin-1 (Kumar et al., 2006), mediating ER extension (Zhang et al., 2010a). However, kinectin associates with ER sheets, and is excluded from axons (Farías et al., 2019). P180 protein, which contains a kinectin-homologous region and two MT-binding domains, also can bind kinesin-1 (Diefenbach et al., 2004), and interestingly, its distribution is not only restricted to the ER cisternae in the soma. P180 is also found at axonal ER tubules, from where it stabilizes MTs (Farías et al., 2019). Similarly, Sec61β, a subunit of the Sec61 translocon complex, is present in both soma and axon (Farías et al., 2019), and interacts directly with MTs (Zhu et al., 2018). Also, REEP1 and spastin can bind MTs, promoting respectively ER alignment along MTs (Park et al., 2010) and MT severing (Roll-Mecak and Vale, 2005). Nevertheless, the potential role for these ER proteins in ER transport remains to be explored.

Axonal ER is mostly composed of interconnected ER tubules, with occasional small sheets or cisternae (Wu et al., 2017; Yalçın et al., 2017). Antagonistically to tubular ER-shaping proteins, ER membrane protein Climp63 promotes formation of ER sheets, stabilizing them through homodimeric luminal bridges (Shibata et al., 2010). Interestingly, Climp63 mediates static interactions with MTs, stabilizing the ER network (Verkhovsky et al., 2005).

#### ER-Shaping Proteins and Tubular ER Motility

Proteins that shape or regulate tubular ER also influence ER tubule motility, although the mechanisms for this are not always clear. For example, Rab10 depletion decreases both extension frequency and fusion efficiency of ER tubules (English and Voeltz, 2013). Rab10 is enriched at the leading edge of nearly half of all dynamic ER tubules, and these dynamic domains track along MTs (English and Voeltz, 2013), suggesting a direct role for Rab10 in regulating tubular ER movements along MTs. VAPA/B knockdown impairs tubular ER movements in axons (Lindhout et al., 2019), potentially by affecting ER MCSs, which may destabilize the ER network. Also, RTN overexpression reduces retraction speed and increases fission frequency of ER tubules (Espadas et al., 2019); since RTN is distributed along the tubular ER network, its role in ER dynamics might simply result from its effect on the physical properties of the ER tubules.

#### ER MCS Dynamics

ER movements also result from transport of ER-associated organelles along MTs. As they traffic, endosomes and mitochondria can keep their MCS with ER, which results in changes in the position and morphology of the tethered ER tubules (Wu et al., 2018). This may be a way to ensure that during traffic events, ER tubules still can regulate these organelles, including their fission, lipid composition, or Ca^2+^ levels. The number and the size of ER MCS are highly dynamic, and dependent on physiological conditions. For example, Ca^2+^ levels can regulate ER-PM lipid exchange through the control of E-Syt tethering and function (Yu et al., 2016; Bian et al., 2017), and also STIM1 activation, promoting STIM1-Orai1 tethering to mediate Ca^2+^ exchange between ER and PM (Carrasco and Meyer, 2011). Key cellular functions have also been associated with ER-mitochondria MCS dynamics. For example, mitochondrial ATP production depends on mitochondrial Ca^2+^ uptake at ER-mitochondria MCSs. The importance of balanced ER-mitochondrial MCSs is illustrated by the fact that excessive mitochondrial Ca^2+^ uptake from ER can turn detrimental for the cell, triggering excessive opening of the mPTP and causing apoptosis (Paillard et al., 2013).

#### ER Turnover

To maintain ER homeostasis, the ER network requires a regulated system to remove excessive ER expansion. In addition, removing ER could facilitate network remodeling. Most of our knowledge about ER autophagy (ER-phagy) is related with the unfolded protein response (UPR), a degradative system that allows the removal of excessive accumulated misfolded and unfolded proteins in the ER lumen (Lindholm et al., 2017), and therefore, mostly associated with rough ER. An additional role for ER-phagy is turnover and clearance of ER (Grumati et al., 2018). This process is regulated by integral ER proteins, which act as ER-phagy receptors, targeting ER fragments to autophagosomes for lysosomal degradation. To date, six different ER-phagy receptors (see below) have been identified. Each of them specifically binds to ATG8 family members, such as LC3 and GABARAP, which are found in the membrane of incipient autophagosomes (Birgisdottir et al., 2013). Consistent with the hypothesis that ER-phagy can be activated locally to regulate ER excess, different ER-phagy receptors are found at different ER network domains. For rough ER, an ER-phagy receptor role has been reported for Sec62, a member of the translocon complex (Fumagalli et al., 2016), and for cell-cycle progression gene 1 (CCPG1), which is activated by the UPR (Smith et al., 2018). Similarly, ER-phagy receptor FAM134B has been linked to turnover of perinuclear ER cisterna (Khaminets et al., 2015; Bhaskara et al., 2019). Remarkably, the molecules identified as tubular ER-phagy receptors are tubular ER-shaping proteins. A long RTN3 isoform, which contains several LC3-interacting regions in its large amino-terminal domain, can promote degradation of ER tubules (Grumati et al., 2017). A similar role for ATL3 has also been observed (Chen et al., 2019). Recently, the predicted single-pass transmembrane ER protein, TEX264, was identified as an ER-phagy receptor (An et al., 2019; Chino et al., 2019), specifically mediating the degradation of ER tubule three-way junctions (An et al., 2019). All these ER-phagy receptors have been studied in non-neuronal mammalian cells, and their roles in neurons remain to be explored. More comprehensive details about ER-phagy process can be found in recent specific reviews (Grumati et al., 2018; Wilkinson, 2019).

## ER and Axonal Neurodegeneration

**FIGURE 4:**
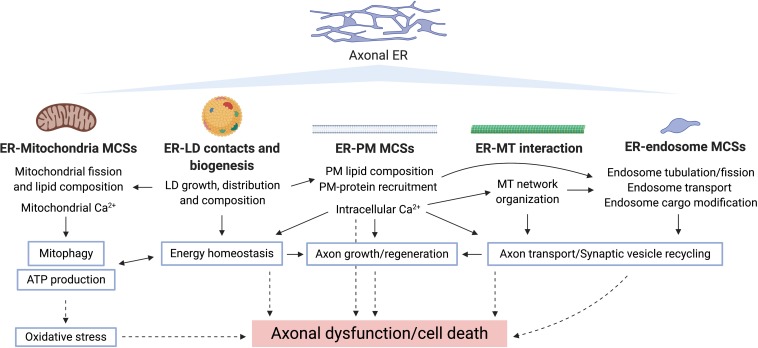
Potential routes underlying axonal ER involvement in neurodegeneration. Bold type shows pathways by which axonal ER may influence other cellular compartments/organelles, where the specific processes regulated in each case are indicated. Blue-border text boxes show processes indirectly regulated by ER. Arrows show regulation of the indicated process. Broken lines show consequences of disrupting the corresponding process.

The physiological roles of ER discussed above make it an obvious potential site of degenerative pathology, for example from impaired lipid, Ca^2+^, or organelle homeostasis. However, its physical properties, in particular its continuity, offer additional vulnerabilities for pathology. A summary of the evidence relating tubular ER and neurological disorders is presented in Table 2. In this section, we will cover the relationship between axonal ER and some neurodegenerative diseases, by looking at causative mutant proteins and their potential roles in disease mechanisms (summarized in Figure 4).

### Hereditary Spastic Paraplegias (HSPs)

The HSPs are a group of inherited, progressive, heterogeneous diseases, predominantly characterized by degeneration of longer upper motor neurons (those with somata in the brain, and axons projecting down the spinal cord). In “pure” HSPs, the main symptoms are lower limb weakness and spasticity. Additional symptoms are observed in “complicated” HSPs, including cerebellar ataxia, peripheral neuropathy, or optic atrophy, among other symptoms. Over 80 distinct spastic gait genetic loci (SPG1-80 and others) are so far known to be causative for HSP. These genes implicate cellular pathways such as membrane shaping and trafficking, mitochondrial function, lipid and protein metabolism, and axon development (Blackstone, 2018; Boutry et al., 2019). Remarkably, around half of HSP patients carry mutations affecting the ER-shaping proteins spastin, ATL1, RTN2 or REEP1 (Boutry et al., 2019), suggesting a relationship between ER modeling and axon maintenance. Other SPG proteins, e.g., REEP2 (Esteves et al., 2014), ARL6IP1, Rab3GAP2 (Novarino et al., 2014), or seipin (Windpassinger et al., 2004) affect ER morphogenesis, and others are also localized on ER (Liberski and Blackstone, 2017), supporting the idea that ER maintenance is essential for axonal maintenance. Mutations in Vps13D, required for mitochondrial size and clearance (Anding et al., 2018), are also causative for recessive spastic paraplegia or ataxia (Gauthier et al., 2018; Seong et al., 2018), thus suggesting that lipid transfer to or from the ER might be vulnerable in HSP, although the exact subcellular compartments where Vps13D acts are still unknown. There is experimental evidence for roles of several ER-localized SPG proteins in neurite or axon outgrowth (Chen et al., 2000; GrandPré et al., 2000; Gil et al., 2012). However, at least in the pure HSPs, axon growth and development must be largely unaffected, and the mechanisms that lead to degeneration are not necessarily the same ones that impair neurite outgrowth. In addition to their roles in tubular ER morphogenesis, some of the ER-shaping proteins related to HSP also have roles in other cellular processes, such as lipid metabolism, Ca^2+^ signaling or regulation of MTs.

Both ATL1 and seipin regulate LD growth (Szymanski et al., 2007; Klemm et al., 2013; Wang H. et al., 2016; Salo et al., 2019). In spite of the little information about LD in axons, they are implicated in membrane biogenesis and cellular signaling. Therefore, their role in axons may be relevant in pathogenesis (Pennetta and Welte, 2018).

REEP1 coprecipitates and co-aligns with polymerized MTs, indicating that it might help to modulate coupling of the tubular ER network with MT dynamics (Park et al., 2010). In cortical motor neuron cell bodies, REEP1 knockout shows reduced complexity of the peripheral ER network, producing a reduced number of ER structures with increased length (Beetz et al., 2013). REEP1 is also found in axonal growth cones (Park et al., 2010), and its knockdown results in neurite outgrowth defects and degeneration (Lim et al., 2015). Moreover, REEP1 is detected at ER-mitochondria MCSs, suggesting that mitochondrial disruption might be due to disrupted tubular ER organization, which affects lipid and Ca^2+^ exchange between ER and mitochondria through contacts (Lim et al., 2015).

Spastin is a MT-severing protein that also has roles in ER-endosome interactions and LD maturation (Papadopoulos et al., 2015; Allison et al., 2017). In zebrafish, knockdown of spastin causes reduced outgrowth in motor neuron axons and connectivity problems, as well as apoptosis in other neuron types (Wood et al., 2006). In an iPSC (induced pluripotent stem cell) model of spastin-linked HSP, axons displayed swellings with accumulation of mitochondria and tau, suggesting axonal transport problems (Denton et al., 2014). Spastin and ATL1 might function in axonal regeneration by mediating amounts of ER and MTs in growing axon tips (Levitan et al., 2016). All these data suggest that ER dynamics is critical in neurodegeneration and affects not only ER functions but also mitochondria, endosomes, and LDs, perhaps through ER MCSs with them.

*ARL6IP1* knockdown results in abnormal branching of spinal motor neuron axons and a curly tail phenotype in zebrafish, suggesting a role for ARL6IP1 in neuronal development (Novarino et al., 2014). In *Drosophila*, knockdown of ARL6IP1, which causes tubular ER loss and elongated mitochondria in presynaptic terminals, also leads to a progressive locomotor deficit (Fowler and O’Sullivan, 2016).

### Warburg Micro Syndrome (WRBM)

WRBM is an autosomal recessive, developmental brain disorder with symptoms including intellectual disability, microcephaly, cataracts in early childhood, and lower limb spasticity (Warburg et al., 1993). Mutations in *Rab18*, *Rab3GAP1*, *Rab3GAP2*, and *TBC1D20* cause WRBM (Bem et al., 2011; Borck et al., 2011; Handley et al., 2013; Liegel et al., 2013). RAB3GAP1 and RAB3GAP2 form a heterodimer that (despite the name) is a guanine nucleotide exchange factor that activates Rab18 (Gerondopoulos et al., 2014), and TBC1D20 has been reported as a GTP-activating protein that inactivates Rab18, suggesting a common disease mechanism involving Rab18 signaling (Handley et al., 2015). These proteins have roles in ER organization, ER-LD tethering, secretion, and autophagy (including in neurons) (Nevo-Yassaf et al., 2012; Liegel et al., 2013; Gerondopoulos et al., 2014; Sidjanin et al., 2016; Xu et al., 2018; Dejgaard and Presley, 2019; Nian et al., 2019). Recruitment of Rab18 to ER by Rab3GAP is essential for a normal ER tubular network, and this is disrupted by disease-causing mutations (Gerondopoulos et al., 2014), making ER a potential site for WRBM pathology. WRBM presents as a complicated spastic paraplegia that progressively spreads to the upper body. Therefore, the paraplegia symptoms could potentially be caused by ER defects as in HSP, with additional symptoms due to other roles of Rab18, Rab3GAP and TBC1D20.

### Alzheimer’s Disease (AD)

Alzheimer’s disease is the most common neurodegenerative disease, causing a decline in memory, difficulties in speaking, writing, understanding, identifying objects, and disorientation. Its main molecular hallmarks are extracellular beta-amyloid plaques, intracellular tau tangles, dystrophic neurites (DNs) and dysfunctional mitochondria (Scheltens et al., 2016; Kumar K. et al., 2018). Mutations in amyloid precursor protein (APP), and in the proteases that generate beta-amyloid from APP, Presenilin-1 (PS1, PSEN1) and Presenilin-2 (PS2, PSEN2) can cause early-onset AD, and apolipoprotein E/E4 (APOE4), a protein implicated in lipid metabolism and inflammation, is a strong genetic risk factor for late-onset AD (Van Cauwenberghe et al., 2016; Kumar K. et al., 2018).

Dysfunctional tubular ER in DNs is a feature of AD pathology (Sharoar et al., 2016). Mutations in *Rab10* are associated with AD (Ridge et al., 2018). Rab10 binds to both MTs and tubular ER (Shim et al., 2008), potentially mediating interaction between them; therefore, proper axonal ER dynamics and transport may be processes that are vulnerable in AD. The tubular ER-shaping proteins RTN3 and RTN4B interact with β-secretase BACE1 (β-amyloid converting enzyme 1) (He et al., 2004), and negatively modulate BACE1 cleavage of APP during release of amyloid β peptides (Shi et al., 2009; Araki et al., 2013). RTN3 but not RTN1 is preferentially localized in DNs (Shi et al., 2017), and transgenic mice that overexpress RTN3 accumulate DNs and show impaired learning and memory and synaptic plasticity (Hu et al., 2007), suggesting that DNs with RTN3 aggregates (RTN3 immunoreactive DNs, or RIDNs) can cause cognitive dysfunction in AD. Additional ER shaping proteins like REEP2 and REEP5 also localize to RIDNs, but not other structural or ER stress-related proteins, suggesting that RIDN formation is due to accumulation of tubular ER in axons (Sharoar et al., 2016). In addition, aging may facilitate RIDN formation; in AD mouse brains, RIDN formation with abnormal ER tubules in axons increases over time (Sharoar et al., 2016).

Alterations in tubular ER network may also affect ER-mitochondria contacts in AD pathology; PS1 and PS2 are highly enriched in ER-mitochondria MCSs (Area-Gomez et al., 2009). In fact, amyloid β production is particularly high at ER-mitochondria MCSs, causing increased ER-mitochondria tethering and related functions such as lipid and Ca^2+^ exchange (Hedskog et al., 2013; Schreiner et al., 2014). Altered ER Ca^2+^ homeostasis is becoming a central player in AD pathology. Beta-amyloid plaques impair neuronal Ca^2+^ homeostasis (Kuchibhotla et al., 2008). Aβ oligomers are capable of forming Ca^2+^ permeable channels in the PM, and this alters Ca^2+^ homeostasis by elevating Ca^2+^ in the cytosol, resulting in cytotoxicity (Demuro and Parker, 2013). Also, wild type PS1/2, independent of secretase activity, can form Ca^2+^-permeable ion channels in ER membrane to leak Ca^2+^ to the cytosol, whereas in PS1/2 mutants the ER is overloaded with Ca^2+^, similar to aging neurons (Tu et al., 2006). In this context, several ER proteins involved in Ca^2+^ signaling (IP3R, RyR, SERCA, SigR1, STIM1, and STIM2) appear related with AD pathogenesis (Table 2), supporting a critical role for ER Ca^2+^ in AD. Also, both AD patients and mouse AD models show decreased MFN2 protein levels, correlating with disruption of mitochondria dynamics (Wang et al., 2009; Manczak et al., 2011), and increasing ER-mitochondria MCSs ameliorates AD defects in a *Drosophila* model (Garrido-Maraver et al., 2019).

### Amyotrophic Lateral Sclerosis (ALS)

Amyotrophic lateral sclerosis is a fatal neurodegenerative disease with upper and lower motor neuron death, leading to progressive weakness and atrophy of muscles and to paralysis (Hardiman et al., 2017). Most ALS cases are sporadic, but some are familial (Beleza-Meireles and Al-Chalabi, 2009). Almost half of the familial cases are due to mutations affecting superoxide dismutase (SOD1), TAR DNA-binding protein 43 (TDP-43), fused in sarcoma (FUS) and dipeptide repeat proteins derived from the *C9ORF72* gene. Another ALS gene encodes VAPB (Nishimura et al., 2004; Ling et al., 2013), which like VAPA, localizes to ER and mediates tethering between ER and other organelles (Murphy and Levine, 2016), and regulates tubular ER morphogenesis and dynamics (Lindhout et al., 2019). Both VAPA and VAPB expression is reduced in ALS patients, SOD1-ALS mice, and in HeLa cells. ALS-causative mutation of VAPB produces accumulation of VAPB in the cytosol and VAPB aggregates that contain ER tubules (still continuous with the ER and linked to mitochondria outer-membrane). Neurons expressing this *VAPB* mutant allele show increased cell death (Teuling et al., 2007). In addition to its association with ALS, the same VAPB mutation has been found in spinal muscular atrophy (SMA) patients (Nishimura et al., 2004). SMA is caused by degeneration of alpha motor neurons in the spinal cord, producing progressive muscle paralysis and atrophy (Tisdale and Pellizzoni, 2015).

ER MCSs appear to be important in ALS pathology. In motor neurons isolated from ALS patients and in the spinal cord of SOD1-ALS mice, a proapoptotic and mitophagy-associated protein BNIP1 (BCL2 interacting protein 1) was found as an ALS risk gene, and the expression level of BNIP1 was lowered compared to controls (Karch et al., 2018). BNIP1 plays a role in ER network organization by mediating ER membrane fusion (Nakajima et al., 2004). Also, mutations in SigR1, a chaperone that regulates ER Ca^2+^ channels (Hayashi and Su, 2007; Srivats et al., 2016), are associated with ALS (Al-Saif et al., 2011; Prause et al., 2013). Ataxin-2, an RNA-binding protein required for proper tubular ER morphogenesis and dynamics (del Castillo et al., 2019), is associated with ALS (Elden et al., 2010). Depletion of ataxin-2 causes disruption of peripheral ER morphology, more tubular instead of cisternal, and abnormal LD structure together with defective mitochondria morphology in *C. elegans* embryos (del Castillo et al., 2019).

### Parkinson’s Disease (PD)

Parkinson’s disease is a common neurodegenerative disorder linked to aging, with symptoms like tremor, rigid muscles, bradykinesia and postural abnormalities. It can be caused by both genetic and environmental factors. A common feature in any form of PD is loss of dopaminergic neurons, which have long unmyelinated axons, with accumulation of intraneuronal Lewy body inclusions (Poewe et al., 2017). Several crucial cellular pathways, such as ER function, protein degradation, Ca^2+^ signaling and intracellular trafficking, have been linked to PD (Cherubini and Wade-Martins, 2017).

Managing Ca^2+^ homeostasis is one of the main roles of tubular ER. Mutation of *PINK1*, a PD causative gene, increases mitochondrial defects such as loss of membrane potential, increased size, and reduced ATP levels, all of which are rescued in PD cell models by inhibition of mitochondria calcium uniporters, which take up Ca^2+^ released from ER (Marongiu et al., 2009). Also, mutations in α-synuclein cause familial dominant forms of PD (Meade et al., 2019); mutant α-synuclein can disrupt ER-mitochondria tethering by binding to VAPB (De Vos et al., 2012; Paillusson et al., 2017). Therefore, ER-mitochondria contacts may potentially affect the severity of PD symptoms, and offer potential strategies for therapy. *LRRK2* (*leucine rich repeat kinase 2*) is another PD causative gene. In astrocytes, mutant LRRK2 localizes to ER membrane where it suppresses activity of the Ca^2+^ ATPase SERCA, causing Ca^2+^ depletion in ER, and overload of Ca^2+^ in mitochondria via induced ER-mitochondria MCSs, eventually resulting in dysfunctional mitochondria (Lee J.H. et al., 2019). This mechanism could potentially also operate in neurons and axons. Mutations in *Vps13C*, encoding a lipid-transfer protein localized at both ER-endosome and ER-LD MCSs (Kumar N. et al., 2018), are associated with PD, and cause mitochondrial dysfunction and PINK1/Parkin-dependent mitophagy (Lesage et al., 2016; Schormair et al., 2018).

Loss of MFN2 results in progressive and retrograde degeneration of dopaminergic neurons in the nigrostriatal circuit in mice, increasing mitochondrial fragmentation and decreasing mitochondrial transport (Pham et al., 2012). MFN2 plays a role in mitophagy mediated by PINK1-Parkin, and knockout of MFN2 in dopaminergic neurons leads to impaired localization of Parkin and axonal loss (Tanaka et al., 2010; Lee et al., 2012; Chen and Dorn, 2013). These data also suggest potential roles of ER and ER tethering in PD mechanisms, as mitophagy occurs via ER-mitochondria physical contacts (Böckler and Westermann, 2014; Puri et al., 2019). Moreover, in *Drosophila* and iPSC PD models, increases in ER-Mitochondria MCSs cause abnormal lipid trafficking, depleting phosphatidylserine from ER. This ER lipid defect impairs sleep patterns (Valadas et al., 2018), one of the non-motor symptoms associated with PD (Munhoz et al., 2015).

MCTP2 gene, a human homolog of Pex30, with potential roles in tubular ER formation (Joshi et al., 2018), was found as a risk factor for early onset PD development (Latourelle et al., 2009), suggesting that disrupted ER organization in dopaminergic axons might facilitate PD development.

### Huntington’s Disease (HD)

Huntington’s disease is an autosomal dominant, progressive, and neurodegenerative disease, with cognitive decline and defects in motor coordination. The cause of the disease is a CAG repeat expansion in the *HTT1* gene, encoding huntingtin, a ubiquitously expressed cytoplasmic protein. Mostly spiny projection neurons (SPNs) degenerate in HD, but it is not clearly known why mutant Huntington (mHTT) protein selectively targets these neurons (Bates et al., 2015). The function of wild type Huntingtin (wHTT) protein is not fully understood, but its multiple protein-protein interaction sites indicate that it might have a scaffolding role (Ochaba et al., 2014).

Disrupted Ca^2+^ signaling is also observed in HD disease models (Mackay et al., 2018), suggesting that disordered Ca^2+^ handling makes SPNs more vulnerable to Ca^2+^-mediated cell death. Ca^2+^ disruption also causes aberrant synaptic plasticity (Hidalgo and Arias-Cavieres, 2016). Binding of mHTT to IP3R increases receptor responsiveness to IP3, resulting in enhanced Ca^2+^ release (Tang et al., 2003), which may ultimately lead to apoptosis. The enhanced Ca^2+^ leak also depletes ER Ca^2+^ stores, triggering SOC replenishment of ER Ca^2+^ (Mackay et al., 2018). This interaction between mHTT and IP3R might be a cause for neurodegeneration. Consistent with this, IP3R blockers prevent enhanced glutamate-mediated cell death in mouse striatal neurons (Tang et al., 2005), and in mouse neuronal cell culture. Moreover, IP3R knockdown prevents synapse loss, and inhibition of SOC channels rescues spine loss (Wu J. et al., 2016). Also, a repeat expansion in the gene encoding JPH3 associates to HD-like 2 (Holmes et al., 2001), which phenocopies HD (Schneider and Bird, 2016). JPH3 promotes ER-PM MCSs, controlling Ca^2+^ communication in hippocampal neurons (Moriguchi et al., 2006). Therefore, ER roles in Ca^2+^ handling appear to be important for HD pathology and could present potential therapeutic targets.

Mutations in Vps13A are causative for chorea-acanthocytosis (Ueno et al., 2001; Shen et al., 2017), a neurological syndrome with a HD-like phenotype (Jung et al., 2011). Since Vps13A is a lipid-transfer protein found at ER MCSs with mitochondria and LDs (Kumar N. et al., 2018), this suggests lipid transfer between ER and other organelles as a primary vulnerability in the disease. However, Vps13A also mediates ER MCS tethering (Kumar N. et al., 2018; Yeshaw et al., 2019), and therefore Vps13A dysfunction could indirectly affect Ca^2+^ communication.

### Peripheral Neuropathies and ER

Peripheral neuropathies are a group of disorders characterized by injury or pathology in the peripheral nervous system (Callaghan et al., 2015). They are heterogenous, with a mix of genetic and acquired causes, clinical presentations, and pathologies. Patients can have motor insufficiency, sensory abnormalities, or both, depending on the disease. Mechanisms implicated in their pathologies include dysregulation of glucose pathway by hyperglycemia, dysfunction of mitochondria, reactive oxygen toxicity, impairments in inflammatory signaling pathways, axonal transport defects, and disrupted K^+^ and Na^+^ channels (Cashman and Höke, 2015). In addition, dysfunctional ER can also contribute. For example, ER stress is one cause of some peripheral neuropathies (Shin et al., 2010; Lupachyk et al., 2013; Inceoglu et al., 2015). Dysfunctional SOC or ER-mitochondria MCSs, which in turn may produce ER stress, can also cause peripheral neuropathies. We will discuss the importance of smooth/tubular ER-associated processes, highlighting the critical role of disrupted Ca^2+^ homeostasis.

Charcot-Marie-Tooth disorder (CMT) [also known as hereditary motor and sensory neuropathy (HMSN)] is one of the most common inherited neurological disorders, with distal weakness and muscle atrophy, presenting with demyelination (CMT type 1) and axonal loss (CMT type 2) (Shy and Patzkó, 2011). CMT type 1A (CMT1A) is a peripheral neuropathy caused by duplications or mutations in peripheral myelin protein 22 (PMP22), a tetra-span membrane protein (Juneja et al., 2019). These lead to demyelination, increased Schwann cell number and axonal loss, although the mechanisms that cause disease are not understood. Recently, it has been found that PMP22 controls Ca^2+^ homeostasis by interacting with the ER membrane protein STIM1, which results in increased Ca^2+^ influx through SOC channels (Vanoye et al., 2019). This supports an important role for ER Ca^2+^ in the pathogenesis of CMT1A, although any role disrupted by PMP mutations would likely be associated with Schwann cells and not with neuronal ER. The peripheral neuropathy CMT2A affects the peripheral and central nervous system, resulting in axon degeneration and progressive sensory loss in patients (Lee et al., 2017). CMT2A occurs as mostly autosomal dominant (Züchner et al., 2004), but sometimes recessive (Iapadre et al., 2018) or semi-dominant (Piscosquito et al., 2015) due to mutations in *MFN2*. As MFN2 localizes at both mitochondrial outer membrane and ER membrane, mediating tethering of both organelles (de Brito and Scorrano, 2008), ER-mitochondria MCSs might be important in the molecular mechanism of CMT2A. In fact, the connectivity between ER and mitochondria correlates with the severity of the disease, and the reinforcement of these contacts prevents some axonal defects caused by MFN2 mutation (Bernard-marissal et al., 2019; Larrea et al., 2019). Neurons and fibroblasts cultured from patients carrying *MFN2* mutations show increased numbers of mitochondria in axons, which appear swollen and degenerating (Verhoeven et al., 2006; Amiott et al., 2008). Moreover, *MFN2* mutant iPSC-derived motor neurons, obtained from CMT2A patients, show disrupted mitochondrial trafficking and cytoskeletal arrangement like patients have, although the mitochondrial morphology is unaffected (Saporta et al., 2015). Therefore, *MFN2* mutations might cause CMT2A pathogenesis through disrupting ER-mitochondria tethering and its functions. This is supported by other recent findings that altered ER-mitochondria MCSs leads to CMT axonopathy (Bernard-marissal et al., 2019), and interestingly, increase of these contact sites promote axon regeneration (Lee S. et al., 2019). Together, this evidence suggests a critical role for ER Ca^2+^ in CMT, particularly in regulating mitochondrial function.

Other tubular ER-related proteins have also been linked to CMT pathologies. The junctophilin JPH1 mediates ER-PM tethering (Golini et al., 2011) and activates RyR channels to release Ca^2+^ from sarcoplasmic reticulum (Hirata et al., 2006). Remarkably, its paralogues JPH3 and JPH4 enable functional coupling of PM Ca^2+^ channels with RyRs in the ER of hippocampal neurons (Moriguchi et al., 2006; Sahu et al., 2019). Deficiency of GADP1 (ganglioside-induced differentiation-associated protein 1), an outer mitochondrial membrane protein associated with CMT, affects Ca^2+^ homeostasis by reducing SOC (Pla-Martín et al., 2013). Interestingly, it has been reported that overexpression of *JPH1* rescues the SOC defects in *GADP1*-silenced cells, and that mutation of both *GADP1* and *JPH1* inhibits SOC activity. In fact, patients where these two genes are mutated show a more severe CMT than those where only *GADP1* is mutated (Pla-Martín et al., 2015). Another example is the ER chaperone SigR1, which modulates the Ca^2+^ release channels IP3R and STIM1 (Hayashi and Su, 2007; Srivats et al., 2016). Mutation in *SigR1* can result in CMT (Li et al., 2015). In addition, mutation of *REEP1* can cause not only HSP, but also spinal CMT, affecting lower motor neurons (Beetz et al., 2012). Mutation of the ER protein seipin is also associated with CMT as well as HSP (Auer-Grumbach et al., 2005). This protein mediates ER-LD tethering (Salo et al., 2016, 2019), and gain-of-toxic-function mutation impairs synaptic neurotransmission (Wei et al., 2014).

Hereditary sensory neuropathies (HSNs) are neurological disorders characterized by degeneration of sensory neurons, with distal sensory loss in the lower limbs. HSNs are classified in eight phenotypically diverse types, which relate to specific genomic mutations and inheritance pattern (Schwartzlow and Kazamel, 2019). Mutations associated to HSNs affect functionally heterogeneous genes, making difficult to find a common pathway affected in patients (Rotthier et al., 2012). Several of the genes associated to the disease directly relate to the ER. Mutations in *FAM134B* can cause HSN type II, which shows autosomal recessive inheritance and manifests at early childhood. FAM134B is found at the *cis*-Golgi network, regulating the morphology of this structure (Kurth et al., 2009), but also at the membrane of perinuclear ER, where acts as a ER-phagy receptor, mediating ER sequestration into autophagosomes (Khaminets et al., 2015). The most common HSN is HSN type I, presenting a progressive autosomal dominant pattern of inheritance and causing a slowly progressive neuronal degeneration (Schwartzlow and Kazamel, 2019). HSN type I can be caused by mutations in *SPTL1* or *SPTL2* genes, which respectively encodes for subunits 1 and 2 of the enzyme serine palmitoyltransferase (SPT). This enzyme is found at the ER membrane, and is required for the synthesis of sphingolipids (Lowther et al., 2012), which present a key role in cell signal transduction (Hannun and Obeid, 2018). However, as for FAM134B, the current evidences do not support SPT distribution at axonal ER, and therefore, it remains unknown whether these proteins may have a role in axonal ER regulation. Other HSN type I-associated mutations affect the tubular ER-shaping proteins ATL1 (Guelly et al., 2011) and ATL3 (Fischer et al., 2014; Kornak et al., 2014). The *ATL3* sensory neuropathy-causing mutation, *ATL3*^*Y192C*^, produces a less profuse ER network and prevents ATL localization at the axonal ER (Behrendt et al., 2019). Moreover, this mutation causes an increased number of ER-mitochondria MCSs, altering mitochondrial dynamics and distribution (Krols et al., 2019). This evidence suggests potential mechanisms by which defects in tubular ER may produce HSN.

Diabetic neuropathies are a type of neurodegenerative disease that mainly target sensory axons, autonomous axons and to some extent, motor axons (Feldman et al., 2019). Common symptoms are numbness and paresthesia, and feet are affected earlier than hands more severely together with autonomic or motor neuropathies (Charnogursky et al., 2014). ER-mitochondria MCSs play an essential role in diabetic neuropathies. They are regulated by glucose levels, acting as a nutrient sensor to switch between fat and glucose oxidation (Theurey and Rieusset, 2017). High glucose levels disrupt both integrity and function of ER-mitochondria MCSs in hepatic cells, inducing mitochondrial fission and impaired respiration (Theurey et al., 2016). This glucose-dependent regulation of ER-mitochondria MCSs is defective in the liver of insulin-resistant obese mice (Theurey et al., 2016), which show increased ER-mitochondria MCSs, causing mitochondrial dysfunction through mitochondrial Ca^2+^ overload (Arruda et al., 2014). Pancreatic beta-cells from patients with type 2 diabetes show reduced ER-mitochondria MCSs, and a similar effect is observed after fatty acid overload (Thivolet et al., 2017). Interestingly, ER-mitochondria MCSs are reduced in pro-opiomelanocortin neurons of diabetic mice, and reduction of ER-mitochondria MCSs in the same neurons, via ablation of *MFN2*, induces leptin resistance and obesity (Schneeberger et al., 2013). In addition, type 1 diabetic rats show reduced ER Ca^2+^ content in DRG neurons, possibly due to decreased levels of the ER Ca^2+^ ATPase SERCA (Verkhratsky and Fernyhough, 2008). Therefore, regulation of Ca^2+^ homeostasis through ER-Mitochondria MCSs is critical in diabetes pathogenesis, including the associated peripheral neuropathy. In rat cortical neurons rats, diabetes-induced neuritic dystrophy and cognitive dysfunction correlate with formation of aggregates of the tubular ER-shaping protein RTN3, similar to that observed in AD (see above). Aggregation of RTN3 prevents its inhibitory effect on BACE1, leading in turn to aggregation of amyloid β and diabetic dementia (Zhao et al., 2013). However, it remains to be explored whether similar defects are associated with diabetes in peripheral nerves.

Human immunodeficiency virus (HIV)-associated sensory neuropathy commonly develops during HIV infection, characterized by numbness and hypersensitivity of the feet and lower legs, and above the knees in more severe cases (Mangus et al., 2014). In primary DRG neurons, HIV coat protein gp120 causes axonal degeneration by inducing rapid large IP3-mediated Ca^2+^ release from ER. Decreasing ER Ca^2+^ storage promotes neuronal resistance to gp120-induced cell death (Höke et al., 2009), suggesting a crucial role for axonal ER.

Chemotherapy-induced peripheral neuropathy is a frequent side effect of chemotherapy (Zajaczkowska̧ et al., 2019). Dysregulation of Ca^2+^ homeostasis, where ER has a key role, contributes to the pain caused by chemotherapy-induced peripheral neuropathy (Siau and Bennett, 2006). Ca^2+^ signaling from ER to mitochondria may have an important role, since a selective SigR1 antagonist reduces hypersensitivity and the proportion of patients developing severe chemotherapy-induced peripheral neuropathy (Bruna and Velasco, 2018).

## Discussion

Neurodegeneration is a complex and multifactorial process causing progressive neurological diseases. The study of genes that influence these conditions provides information about the biology of neurons and their pathologies, and is a catalyst for development of therapeutic strategies. An increasing body of evidence implicates dysfunctional ER in many neurological disorders that affect axons. In rough ER, ER stress is caused by accumulation of misfolded/unfolded proteins, which activates UPR to restore homeostasis, but if not resolved, causes neuronal degeneration and death (Hetz and Saxena, 2017). However, axonal ER, which is mostly tubular and smooth and undertakes very little protein export, is also linked to neurodegeneration. Tubular ER is directly involved in the regulation of many cellular processes, such as lipid biosynthesis and degradation, Ca^2+^ signaling, or organelles and MT dynamics. In axons, ER forms a mostly tubular network that is pervasive and continuous, and in human axons up to 1 m in length, physically linking the cell body with presynaptic terminals. Therefore, neuronal cells are particularly vulnerable to suffer defects when tubular ER morphology or tubular ER MCSs goes wrong. Mutations in genes encoding both tubular ER-shaping proteins and proteins related with ER MCSs, are observed in patients with different disorders (i.e., HSP, CMT, WRBM, PD, AD) (Table 2), where different types of neurons are preferentially affected. Interestingly, mutations affecting ER morphology may indirectly cause altered ER MCSs, potentially explaining why mutations in genes related to either ER morphology or ER MCSs produce similar neuronal defects. The pervasive and continuous nature of the ER suggests an important role for tubular ER continuity and/or local presence in axons. Evidence also suggests a critical role for ER Ca^2+^, particularly in regulating mitochondrial function. Defects in ER Ca^2+^ handling are associated with the pathogenic mechanisms of different neurological diseases (Table 2), and some of these ER Ca^2+^ defects relate to ER-mitochondria MCSs.

Tubular ER shows a highly dynamic nature, and the transport of ER tubules depends on the MT network. Therefore, it is not surprising that long cells, as neurons are, appear preferentially affected in patients with mutations in genes related with tubular ER dynamics. Therefore, proper axonal ER dynamics (including ER MCSs) and transport may be crucial to managing local changes/responses within the neuron.

Axonal ER has been largely neglected as an organelle since its early characterization around four decades ago. We are only starting to learn about its biological importance, and there is growing evidence for it as a potential site of pathology, and hence a potential therapeutic target, particularly for neurological disorders that affect distal longer axons. The preferential susceptibility of distal axons to disease-causing mutations in ER-shaping proteins points to functional importance of its specialized architecture. Exciting areas for study in the coming years include both the mechanisms that adapt the dynamic tubular ER network to the dimensions of axons – its physical continuity, narrow lumen, density of tubules in the cytosol – and the physiological roles of these architectural features, and how impairment of these functions can lead to degeneration. The genetics of the HSP diseases has opened windows both on possible disease mechanisms, and a fascinating area of basic biology. Understanding the basic biology of axonal ER will lead to new insights on the mechanisms of axon degeneration, and conversely understanding the disease mechanisms will lead to new insights in basic biology of axonal ER. Studies of the basic cell biology and the disease mechanisms will feed each other over the coming years.

## Author Contributions

ZÖ wrote the first draft of the manuscript and designed the figures. CO’K helped draft and edit the manuscript. JP-M wrote the first draft, edited the manuscript, and designed and created the figures.

## Conflict of Interest

The authors declare that the research was conducted in the absence of any commercial or financial relationships that could be construed as a potential conflict of interest.
